# Tissue-Specific Suppression of Thyroid Hormone Signaling in Various Mouse Models of Aging

**DOI:** 10.1371/journal.pone.0149941

**Published:** 2016-03-08

**Authors:** W. Edward Visser, Cíntia R. Bombardieri, Chantal Zevenbergen, Sander Barnhoorn, Alexandre Ottaviani, Ingrid van der Pluijm, Renata Brandt, Ellen Kaptein, Ramona van Heerebeek, Hans van Toor, George A. Garinis, Robin P. Peeters, Marco Medici, Willy van Ham, Wilbert P. Vermeij, Monique C. de Waard, Ronald R. de Krijger, Anita Boelen, Joan Kwakkel, John J. Kopchick, Edward O. List, Joost P. M. Melis, Veerle M. Darras, Martijn E. T. Dollé, Gijsbertus T. J. van der Horst, Jan H. J. Hoeijmakers, Theo J. Visser

**Affiliations:** 1 Dept of Internal Medicine, Erasmus Medical Center, Rotterdam, The Netherlands; 2 MGC Dept of Genetics, Cancer Genomics Center, Erasmus Medical Center, Rotterdam, The Netherlands; 3 Institute for Research on Cancer and Aging, Nice (IRCAN), UMR 7284 CNRS U1081 INSERM UNS, 28 avenue de Valombrose Faculté de Médecine, Nice, France; 4 Laboratory of Comparative Endocrinology, Biology Department, KULeuven, Leuven, Belgium; 5 Dept of Pathology, Erasmus Medical Center, Rotterdam, The Netherlands; 6 Dept of Endocrinology and Metabolism, Academic Medical Center, Amsterdam, The Netherlands; 7 Dept of Biomedical Sciences, Edison Biotechnology Institute, Ohio University, Athens, Ohio, United States of America; 8 Dept of Toxicogenetics, Leiden University Medical Center, Leiden, The Netherlands; 9 Centre for Health Protection Research, National Institute of Public Health and the Environment (RIVM), Bilthoven, The Netherlands; National Cancer Institute, UNITED STATES

## Abstract

DNA damage contributes to the process of aging, as underscored by premature aging syndromes caused by defective DNA repair. Thyroid state changes during aging, but underlying mechanisms remain elusive. Since thyroid hormone (TH) is a key regulator of metabolism, changes in TH signaling have widespread effects. Here, we reveal a significant common transcriptomic signature in livers from hypothyroid mice, DNA repair-deficient mice with severe (*Csb*^*m/m*^*/Xpa*^*-/-*^) or intermediate (*Ercc1*^*-/Δ-7*^) progeria and naturally aged mice. A strong induction of TH-inactivating deiodinase D3 and decrease of TH-activating D1 activities are observed in *Csb*^*m/m*^*/Xpa*^*-/-*^ livers. Similar findings are noticed in *Ercc1*^*-/Δ-7*^, in naturally aged animals and in wild-type mice exposed to a chronic subtoxic dose of DNA-damaging agents. In contrast, TH signaling in muscle, heart and brain appears unaltered. These data show a strong suppression of TH signaling in specific peripheral organs in premature and normal aging, probably lowering metabolism, while other tissues appear to preserve metabolism. D3-mediated TH inactivation is unexpected, given its expression mainly in fetal tissues. Our studies highlight the importance of DNA damage as the underlying mechanism of changes in thyroid state. Tissue-specific regulation of deiodinase activities, ensuring diminished TH signaling, may contribute importantly to the protective metabolic response in aging.

## Introduction

Aging is a complex and still poorly understood process although damage to macromolecules is considered to be an important contributing factor. Particularly, damage to nuclear DNA, which carries the blueprint of life, can exert harmful, widespread and lasting effects on cell function, viability and growth. A causative role of DNA damage in aging is unequivocally demonstrated by the identification of genetic defects in DNA repair in a large fraction of progeroid syndromes [1]. A major route of DNA repair is nucleotide excision repair (NER), a multi-step “cut-and-patch” repair mechanism which is responsible for removing a remarkable diversity of helix-distorting lesions caused by exogenous factors or endogenous metabolism, thus integrating environmental and intrinsic contributions to the aging process.

Important insights into the link between DNA damage and aging have come from studies using rapidly aging mice with defects in transcription-coupled NER (TC-NER) as models for human progeroid syndromes [2–4]. In the absence of TC-NER, the vital process of transcription is hampered by persisting DNA lesions, causing accelerated cell death and thus premature aging. Since progeroid mutants display varying extents of accelerated aging in different tissues, presumably reflecting tissue-dependent induction of DNA damage, expression of repair and/or damage response genes they are termed ‘segmental’ progeroid syndromes. Nevertheless, TC-NER deficient patients and corresponding mouse mutants are valuable models that manifest many genuine aspects of normal aging in an accelerated manner [5–7].

The stochastic accumulation of DNA damage appears to contrast the prominent genetic component also identified in the process of aging. Mutations in specific endocrine signaling pathways, specifically those suppressing the growth hormone/insulin-like growth factor 1 (GH/IGF1) axis, markedly prolong lifespan in numerous organisms including mammals [8], whereas conversely over-expression of GH in transgenic mice accelerates many features of aging and reduces life expectancy [9].

This paradox of genetic regulation of longevity *versus* stochastic damage accumulation was reconciled by studies in progeroid repair mouse mutants [3,4,10]. Genome-wide expression profiles in livers of progeroid and naturally aging mice showed marked similarities, indicating the relevance of progeroid models for normal aging. Interestingly, a suppressed GH/IGF1 axis and concomitantly up-regulated anti-oxidant defenses were observed in short-lived repair mutants, long-lived mice (dwarf mutants with pituitary dysfunction), in normal mice upon chronic non-toxic exposure to DNA-damaging agents, as well as in cultured cells after induction of persisting DNA damage. A similar response is also reported during dietary restriction and in naturally aged mice [3,4, 11], indicating its universal nature. Therefore, the paradox of somatotroph axis suppression in long-lived as well as short-lived animals indicates that this response to accumulating DNA damage represents an attempt to lower metabolism and boost defenses, entailing a shift from growth and development to a preservation mode, which intends to promote lifespan extension [12]. In a wider perspective, suppression of the GH/IGF1 axis which is observed in stressful situations (e.g. food shortage, severe illness) may be regarded as an adaptive ‘survival’ response which transiently prioritizes defense systems at the expense of growth. Because of chronic stress a similar response may become constitutively induced in later stages of the aging process [12].

Since thyroid hormone (TH) is one of the few known genuine stimulators of metabolic rate, changes in TH metabolism and action have widespread effects on the function of organisms ranging from small non-vertebrates to humans. TH is crucial for development and metabolism of virtually all tissues. TH action involves the production of the prohormone T4 by the thyroid gland and its conversion to bioactive T3 in peripheral tissues by the deiodinases D1 and D2. The deiodinase D3 degrades T4 and T3 to receptor-inactive metabolites [13]. Time- and tissue-specific expression of the different deiodinases ensures a coordinated spatiotemporal control of intracellular TH concentrations [13]. Although non-genomic actions have been reported, the biological actions of TH are largely mediated by binding of T3 to nuclear receptors (T3-receptors; TRs), thus modulating the expression of TH target genes. Changes in thyroid state which are associated with aging are likely to have important consequences. For example, dwarf mice with pituitary hormone deficiencies resulting in low GH/IGF1 and TH levels have a prolonged lifespan, and life-long T4 substitution of those animals markedly shortens their lifespan [14–16], suggesting a strong connection of TH with lifespan regulation.

Currently, the molecular mechanisms underlying changes in thyroid state during aging are unknown. Furthermore, it is still elusive if DNA damage contributes to the age-related changes in TH homeostasis and if changes in TH signaling are maladaptive or represent a beneficial response.

Here, we studied TH signaling with a focus on deiodinase regulation in different NER-deficient mice including *Csb*^*m/m*^*/Xpa*^*-/-*^ double mutant mice with extremely accelerated aging (XAA; lifespan 3–5 weeks) and *Ercc1*^*-/Δ-7*^ mice with moderately accelerated aging (MAA; lifespan 4–6 months), and also naturally aging wild-type (WT) mice. We observed that TH signaling is suppressed in accelerated and normal aging. Interestingly, we noticed a dramatic reactivation of the TH-inactivating enzyme D3 in the liver, which is normally mainly expressed in embryonic tissues. Also, studies in animals with an interrupted GH/IGF1 axis (without defects in DNA damage repair) suggest that the D3 induction is independent of the somatotrophic axis. WT mice treated with the pro-oxidant bis(2-ethylhexyl)phthalate (DEHP) also display induction of D3 and suppression of TH signaling. The present findings disclose a novel link between DNA damage, compromised genome maintenance, and TH homeostasis through deiodination. We hypothesize that adaptation of TH signaling has a net beneficial impact on aging.

## Materials and Methods

### Animals

The generation and characterization of NER-deficient *Csb*^*m/m*^*/Xpa*^*-/-*^ and *Ercc1*^*-/Δ-7*^ mice has been previously described [4,17]. With the exception of *Ercc1*^*-/Δ-7*^ mice which were generated in a genetically uniform FVB:C57BL/6J (50:50) F1 hybrid genetic background, all mice were bred in a pure C57BL/6J genetic background. Animals were kept on a regular diet and housed at the Animal Resource Center (Erasmus University Medical Center) and the National Institute of Public Health and the Environment (RIVM), which operate in compliance with the “Animal Welfare Act” of the Dutch government, using the “Guide for the Care and Use of Laboratory Animals” as its standard. *Csb*^*m/m*^*/Xpa*^*-/-*^ and *Ercc1*^*-/Δ-7*^ mice have a lifespan of 3–4 and 22–28 weeks respectively. As required by Dutch law, formal permission to generate and use genetically modified animals was obtained from the responsible local and national authorities. All animal studies were approved by the Animal Ethical Committee of the Erasmus University Medical Center (Dutch equivalent of the IACUC). The generation and characterization of transgenic mice overexpressing bovine growth hormone (bGH TG mice) have been previously reported [18]. Mice deficient in the GH receptor (*Ghr* KO) have been described before [19]. All experiments were conducted in accordance with NIH and local animal care guidelines. Studies were done with mice from both sexes, except for the naturally aged mice (only males) and the DEHP-treated mice (only females). Animals were sacrificed by cervical dislocation.

### Microarray analysis

Independent datasets on liver samples were collected from previous studies [4,20]: 15-day-old *Csb*^*m/m*^*/Xpa*^*-/-*^ (n = 3), 16-week old *Ercc1*^*-/Δ-7*^ (n = 3), and age-matched WT controls (n = 3), 13-week old and 130-week old mice (n = 3) (E-MEXP-835, E-MEXP-1503 and E-MEXP-1504 all based on Affymetrix Mouse Genome 430 2.0 arrays) and euthyroid and hypothyroid WT mice (n = 3) (GSE10001, based on Affymetrix Mouse Gene 1.0 ST arrays). CEL files from these experiments were processed independently using the Partek software and differential expression values and corresponding p-values were obtained using ANOVA. Since the type of arrays used in these studies were different, they were matched by their corresponding RefSeq transcripts IDs (18440 matches in total) using home-made programs (Python^™^). The data was then filtered based on thresholds of ≥1.2-fold change (up and down) and p-values≤0.05.

Venn diagrams were produced using the Venny online tool (http://bioinfogp.cnb.csic.es/tools/venny/index.html) [21]. Significance of the overrepresentation of common and co-directionally regulated genes was approximated using Poisson and binomial distributions.

To analyze for gene expression similarities, we subsequently filtered out genes that were not present in all four data sets. These groups were subsequently clustered based on co-directionality of expression changes and heatmaps were constructed using the MultiExperimentViewer software.

All gene lists were also analysed using the Ingenuity Pathway Analysis software to look for dataset significant enrichment for specific pathways.

### Histology

Thyroid glands from 15-day-old *Csb*^*m/m*^*/Xpa*^*-/-*^ (n = 4), 16-week old *Ercc1*^*-/Δ-7*^ (n = 4) and age-matched WT controls (n = 4) were fixed in formalin and embedded in paraffin, sectioned and stained with haematoxylin/eosin (H&E). All samples were examined blindly.

### Serum and tissue T4 and T3 concentrations

Serum T4 and T3 levels were determined by radioimmunoassay (within- and between assay variability 2–8% and 5–10% for T4 and 2–6% and 8% for T3) as previously described [22]. Briefly, 125I-labeled iodothyronines were obtained from Amersham Pharmacia Biotech. T4 antiserum was obtained from Sigma-Aldrich (St. Louis, MO), and T3 antiserum was produced in the Rotterdam laboratory. Final antibody dilutions were 1:20,000 for T4 and 1:250,000 for T3. The sample volume was 10 μl for T4, 20 μl for T3, and incubation mixtures were prepared in 0.5 ml RIA buffer. Mixtures were incubated in duplicate overnight at 4 C, and antibody-bound radioactivity was precipitated using Sac-Cel cellulose-coupled second antibody (IDS, Boldon, UK). Tissue T4 and T3 content was measured by radioimmunoassay after extraction, as previously described [23]. Briefly, RIAs were used for the determination of T4 and T3 in tissues but prior to the RIA the THs were extracted. The sample was ho- mogenized directly in methanol, and [131I]T4 and [125I]T3 were added to each sample as internal tracers for recovery calculations. These tracers were added in amounts small enough to avoid interferences in the final RIAs. Appropriate volumes of chloroform were added to extract with chloroform/methanol (2:1), twice. The iodothyronines were then back-extracted into an aqueous phase and purified by passing this aqueous phase through Bio-Rad AG 1x2 resin columns. After a pH gradient, the iodothyronines were eluted with 70% acetic acid, which was then evaporated to dryness and the residue dissolved in RIA buffer. Each extract was extensively counted to determine the recovery of the [131I]T4 and [125I]T3 added to each sample during the initial homogenization process. Concentrations were then calculated using the amounts of T4 and T3 found in the respective RlAs, the individual recovery of the [131I]T4 and [125I]T3 added to each sample during the initial homogenization process, and the weight of the tissue sample submitted to extraction. The results are given throughout in picomoles per gram wet weight. Serum TSH levels were determined as described previously [24].

### Deiodinase activities

The activities of the deiodinases D1, D2 and D3 were measured as reported previously [25]. Briefly, tissues were homogenized in 10 vol 0.1 M phosphate (pH 7.2), 2 mm EDTA, and 1 mm dithiothreitol (P100E2D1 buffer). Aliquots of homogenates were snap-frozen and stored at −80 C until analysis of enzyme activities. Deiodinase activities were assayed by monitoring the preferred reaction catalyzed by the different isoenzymes, i.e. ORD of rT3 by D1, ORD of T4 by D2, and IRD of T3 by D3. D1 and D2 activities were assayed by measurement of the release of radioiodide from outer ring-labeled substrates, and D3 activity was assayed by HPLC analysis of the formation of radioactive 3,3′-T2 and 3′-iodothyronine (3′-T1) from outer ring-labeled T3.

### Oxidative stress

To investigate oxidative stress, 10-week old WT animals were exposed through food to the non-carcinogenic dose of 6000 ppm DEHP daily for a period of 2, 12 or 39 weeks.

### qRT-PCR

cDNA was synthesized using 1 μg RNA and Superscript^™^IIReverse Transcriptase (Life Technologies, Breda, The Netherlands). SYBR Green I (Eurogentec, Maastricht, The Netherlands) was used as detector dye for qRT-PCR of the *Abca3*, *Abcc3*, *Cs*, *Fmo3*, *Elolv3*, *Cbr1*, *Cs*, *G6pc*, *Mup2*, *Nudt7*, *Prodh*, *Slc22a7*, *Stat5b*, *Thrsp* (*Spot14)*, *Dio1*, *Dio3*, *Serca1*, *Serca2*, *Ucp3*, *Slc2a4*, *Glut4*, *Hcn2*, *Myh6 (MHC-α)*, *Myh7 (MHC-β)*, Mct8, Mct10, Ntcp, Lat1, Lat2, Oatp1a1, Oatp1a4, Oatp1b2, Thra and Thrb. genes. Primer sequences are provided in S1 Table. The housekeeping genes *B2M*, *RPS9* and tubulin were used for normalization.

## Results

### Gene expression changes indicate suppression of TH signaling

**Fig 1:**
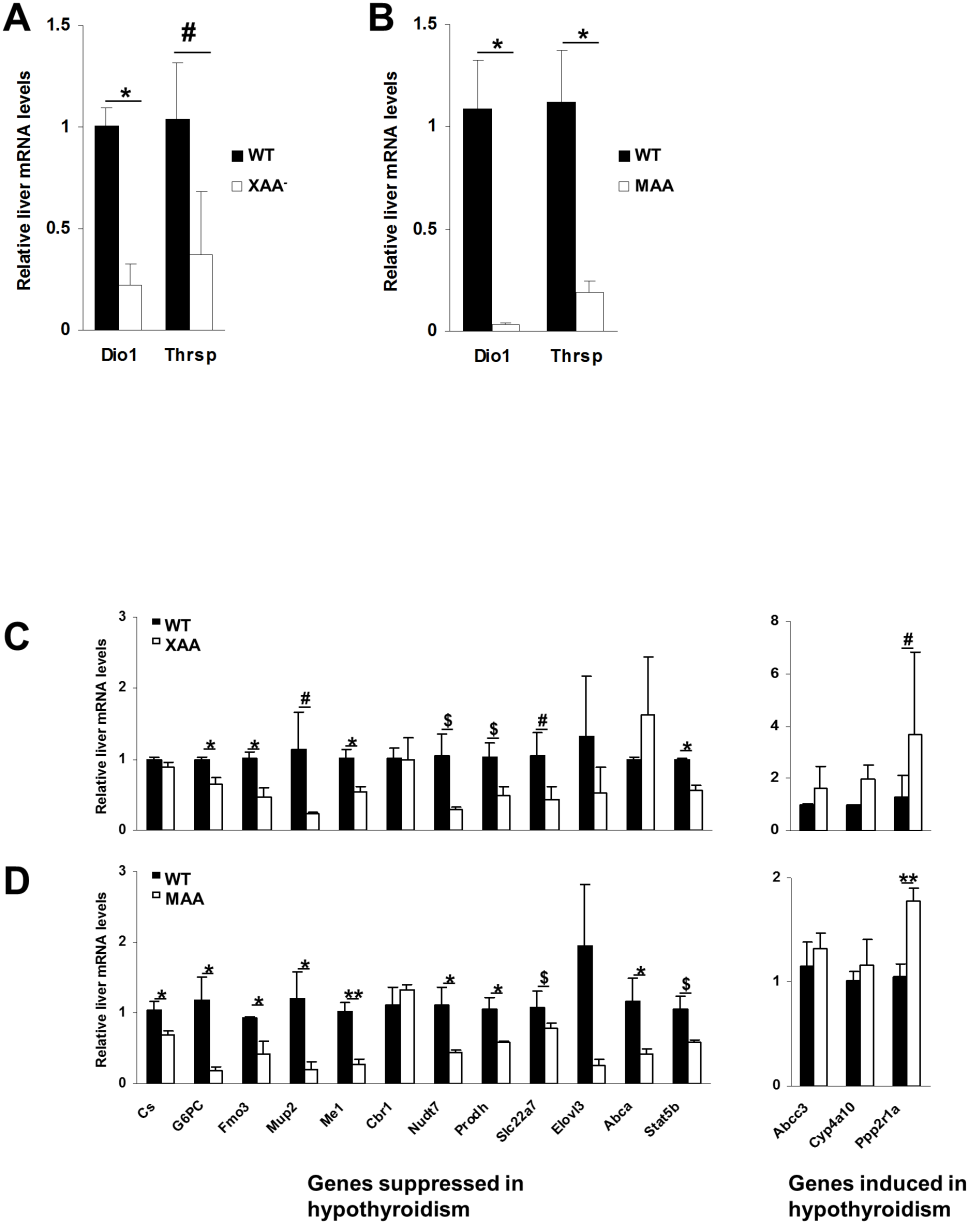
Gene expression changes in livers from progeroid and normal aging mice. Gene expression of the T3-responsive genes Dio1 and Thrsp in 18-day-old XAA (Csbm/m/Xpa-/-) (A) and 16-week-old MAA (Ercc1-/Δ-7) mice (B). * P < 0.005; # P = 0.12. Expression profiling of a set of known T3-responsive genes in 15-day-old XAA (Csbm/m/Xpa-/-) (C) and 16-week-old MAA (Ercc1-/Δ-7) mice (D) mutants compared to age-matched controls. Values represent mean ± SE * P < 0.05; ** P < 0.005; $ P < 0.1; # P = 0.12.

To determine if TH signaling is altered during aging in our mouse mutants, we assessed thyroid state in livers of extremely aging (XAA; abbreviation for *Csb*^*m/m*^*/Xpa*^*-/-*^ KO mice), moderately aging (MAA; abbreviation for *Ercc1*^*-/Δ-7*^ KO mice) and naturally old WT mice. First, we explored if livers of progeroid mice are hypothyroid by measuring expression levels of classic T3-responsive genes. To this aim, we quantified *Dio1* and *Thrsp* mRNA levels, highly sensitive markers of hepatic thyroid state. Expression of both genes was significantly (~3 to 30-fold) decreased in livers of 18-day old XAA mice and 16-week old MAA mice compared to age-matched controls, strongly suggesting that livers of these progeroid mutants are in a hypothyroid state (Fig 1A and 1B).

To obtain a complete overview, we explored if TH-dependent transcriptional programs are altered during aging by genome-wide expression profiling. To this aim we first determined the effect of thyroid state on the liver mRNA expression profile by comparing livers from young adult (9-week old) euthyroid WT mice and WT mice rendered hypothyroid using a low iodine diet supplemented with propylthiouracil [26]. Using predefined criteria, we filtered out a set of 6897 genes which is differentially expressed under hypothyroid conditions (data not shown). In the same way, we compared the microarray datasets of liver samples from 15-day old XAA mice or 16-week old MAA mutants with age-matched WT animals, and from 130-week *versus* 13-week old WT mice, resulting in 3005, 3559 and 2536 differentially expressed genes, respectively. Spearman’s rank correlation coefficients indicate strong similarities between progeroid mutants and naturally aging mice, further supporting the validity of the progeroid models for normal aging ([20] and data not shown). The overlap of differentially expressed transcripts among the various models versus their corresponding controls is represented by a Venn diagram in S1 Fig.

Then, using the list of genes differentially expressed in hypothyroid animals, we asked if there is overlap with the 364 differentially expressed genes common to the naturally aging mice and prematurely aging models. Among these 364 age-related genes, 160 showed a significant change in expression in hypothyroid livers (*p*<0.021, S1 Fig). Approximately 2/3 of these genes displayed a change in the same direction in both hypothyroid and aging profiles (S1 Fig, *p*<0.0001). The set of 160 genes overlapping between hypothyroid and aging mice is presented in S2 Table. It should be realized that the hypothyroid animals were exposed to a low iodine diet supplemented with propylthiouracil which may elicit a response by itself. Hence, in view of the difference in treatment and biological age it is striking that the majority of genes displayed a similar directional change.

Pathway analysis revealed a series of shared biological processes which are over-represented among the differentially expressed genes (up and down) common to hypothyroid and aging mouse models (S2 Fig). This includes a strong representation of the RAR, PPAR/RXR/TR and PI3K/AKT, PTEN signaling systems as well as the ERK/MAPK and other signaling pathways, various cancer-related mechanisms (particularly in the hypothyroid and naturally aging profiles), and components of inflammatory processes. Also the profiles list the involvement of the NRF2-controlled anti-oxidant system and P53 signaling pointing to a link with DNA damage and oxidative stress. Although anticipated, the significant over-representation of the TR/RXR pathway supports the idea of altered TH signaling during both normal and accelerated aging.

As a complementary approach, we measured expression levels of known T3-responsive genes using qRT-PCR. This set of 15 genes was compiled from different independent studies investigating the effect of T3 on the mouse liver transcriptome [27–30]. Indeed, most T3-responsive genes were changed in livers of 15-day-old XAA mice and even more pronounced in 16-week old MAA mice (Fig 1C and 1D). This agrees with the regulation of this set of genes in normally and prematurely aging animals although less pronounced (S3 Fig). Indeed, using qRT-PCR, we observed a trend in reduction in many of these genes, although it failed to reach statistical significance (S3 Fig).

Altogether, gene expression analysis of livers of normally and prematurely aging mice revealed marked similarities with hypothyroid livers, indicating a suppression of TH signaling. These findings support the link between DNA damage, aging and altered TH signaling.

### Thyroid gland histology and serum T4 and T3 concentrations

**Fig 2:**
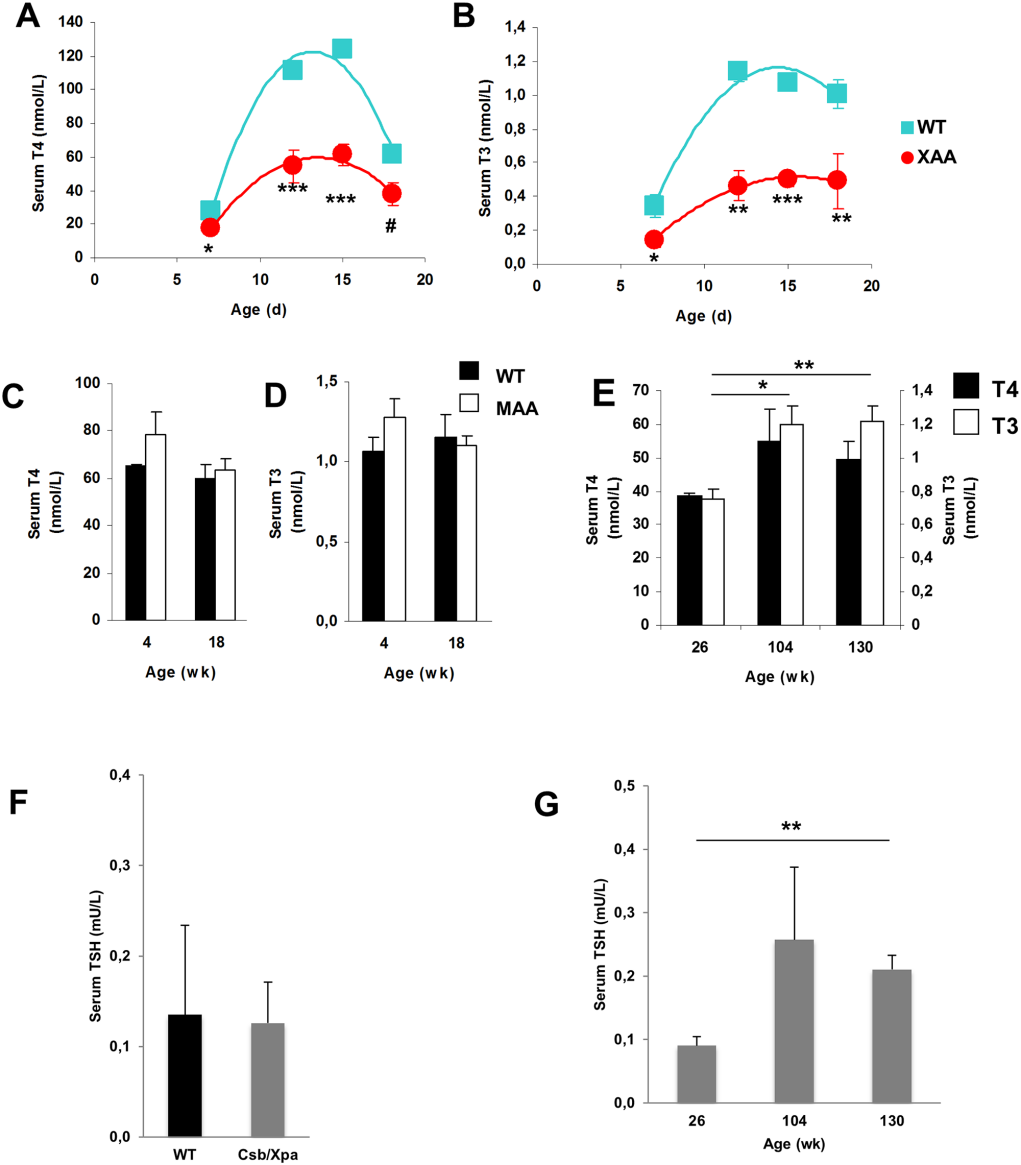
Thyroid state in serum of progeroid and naturally aged mice. Serum T4 (A) and T3 (B) concentrations in 7-, 12-, 15-, and 18-day-old WT (squares) and XAA (Csbm/m/Xpa-/-) mice (circles) (n = 3/group). Serum T4 (C) and T3 (D) concentrations in 4-, and 18-week-old WT (black bars) and MAA (Ercc1-/Δ-7) (white bars) mice (n = 3/group). Serum T4 and T3 concentrations in 26-, 104-, and 130-week-old WT male mice (n = 3-4/group) (E). Serum TSH levels in 15-day old WT and XAA (Csbm/m/Xpa-/-) mice (F) and in 26-, 104-, and 130-week-old WT male mice (G). Values represent mean ± SE per group. * P < 0.05; ** P < 0.01; *** P < 0.001; # P = 0.054.

To investigate the origin of the reduced TH signaling in liver of normally and prematurely aging mice, we first measured serum TH concentrations over time. Significantly lower serum T4 and T3 levels were observed in XAA mice compared to WT controls at all ages examined (Fig 2A and 2B). The differences were already detectable at day 7 and most pronounced at days 12 and 15. The postnatal increase in serum T4 and T3 levels of WT animals, peaking at day 15, perfectly replicated previous findings [22]. Remarkably, although the gene expression profiles of both progeroid mutants suggested an altered thyroid state, serum TH levels did not differ between MAA mice and their normal littermates, at least at the 4 and 18 week time points analysed (Fig 2C and 2D). In addition, no decrease in serum TH concentrations was observed with advancing age in WT animals (Fig 2E). TSH levels were normal in XAA (Fig 2F) and MAA mutants (S4 Fig). A slight increase in TSH levels was noted in 130-week old WT mice (Fig 2G).

**Fig 3:**
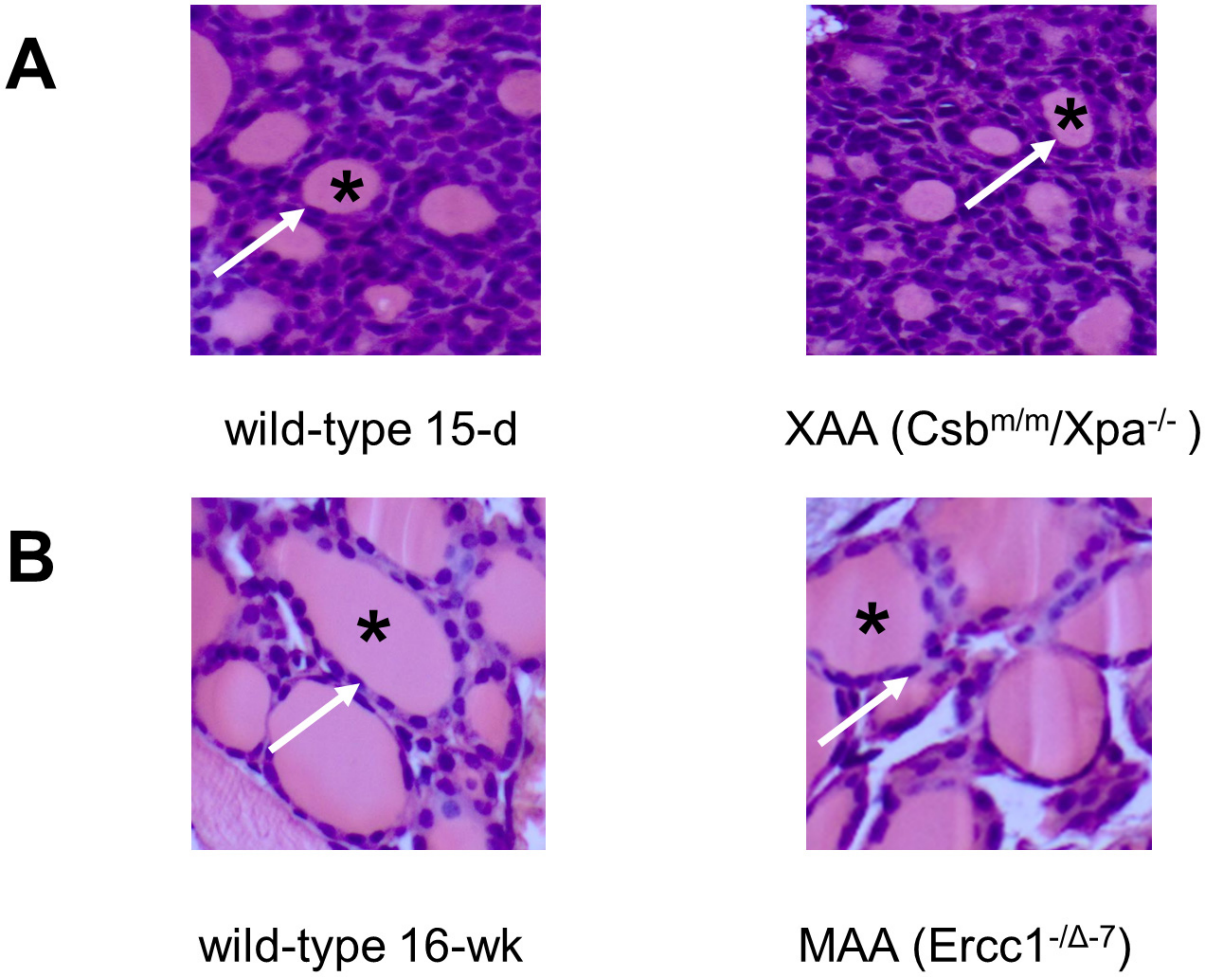
Histological examination of haematoxylin/eosin-stained thyroid glands of 15-day-old WT and XAA (Csbm/m/Xpa-/-) (A) and 16-week-old WT and MAA (Ercc1-/Δ-7) (B) mice (all magnifications 10x). The thyroid follicles (denoted by asterisk) surrounded by thyrocytes (denoted by arrow) are similar between WT and progeria models.

Macroscopic inspection and histological analysis of the thyroid glands did not reveal obvious abnormalities in XAA (Fig 3A) and MAA (Fig 3B) mice. Together, these data suggest that peripheral TH metabolism is altered rather than primary dysfunction of the thyroid gland or pituitary.

### Thyroid state in liver and kidney

**Fig 4:**
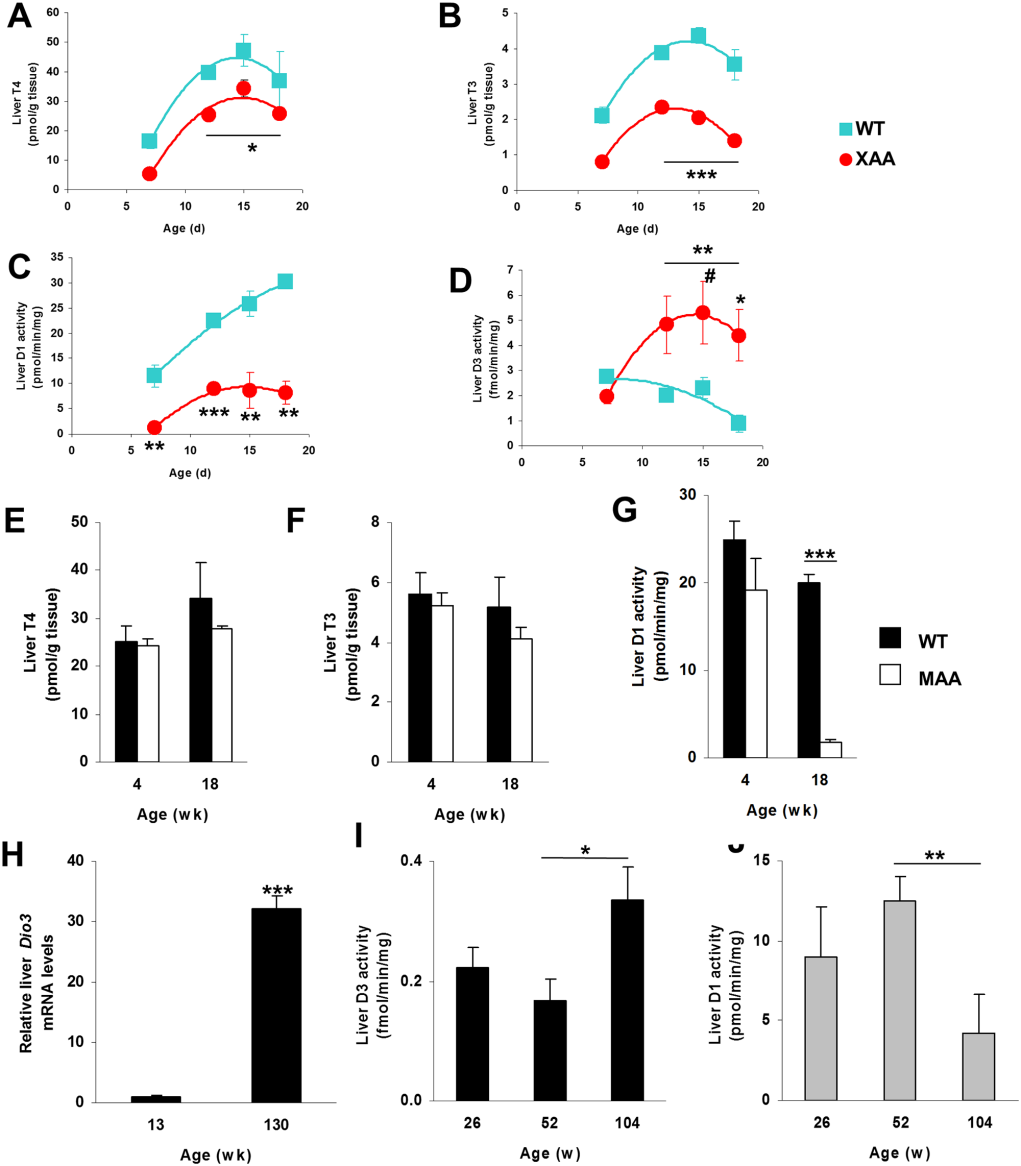
Thyroid state in liver of progeroid and naturally aged mice. T4 (A) and T3 (B) concentrations in livers of 7-, 12-, 15-, and 18-day-old WT (squares) and XAA (Csbm/m/Xpa-/-) (circles) mice (n = 3/group; each time point of Csbm/m/Xpa-/- mice represents pooled tissues). Activities of D1 (C) and D3 (D) in livers of 7-, 12-, 15-, and 18-day-old WT and XAA (Csbm/m/Xpa-/-) mice (n = 3/group). T4 (E) and T3 (F) concentrations and D1 activity (G) in livers of 4-, and 18-week-old WT (black bars) and MAA (Ercc1-/Δ-7) (white bars) mice (n = 3/group). D3 mRNA expression in livers of 13-week-old and 130-week-old mice (H). D1 (black bars) (I) and D3 (grey bars) (J) activities in livers of 26-, 52-, and 104-week-old WT mice (n = 5/group). Values represent mean ± SE per group. * P < 0.05; ** P < 0.01; *** P < 0.001; # P = 0.051.

It is increasingly recognized that local TH concentrations do not necessarily parallel serum TH levels but that tissue TH levels are modulated by deiodinases and transporters [13,31]. Therefore, we determined tissue levels of T4 and T3 in the different mouse models. At all ages tested, hepatic TH levels were lower in XAA mice than in WT mice, although the differences were clearly greater for T3 than for T4 (Fig 4A and 4B). This was reflected in a markedly decreased T3/T4 ratio (0.059± 0.004 in XAA mice *vs*. 0.10±0.01 in WT mice; *P*<0.05). We evaluated the effects of the decreased TH state in liver on D1 activity, which catalyzes T4 to T3 conversion but is also positively controlled by T3. As anticipated, D1 activity increased from postnatal day 5 to 18 in WT mice but much less so XAA mice (Fig 4C).

Since D3 activity may also affect local TH availability, we investigated if the larger decrease in liver T3 than in serum T3 levels is explained by an increased D3 activity. Interestingly, a large induction of D3 activity was observed in XAA mice (Fig 4D). D3 activity was already stimulated at day 12, preceding the drop in liver T3 content at day 15, suggesting that the increased D3-mediated T3 degradation aggravates the reduced T3 concentrations. It is important to note that XAA mice are not yet cachectic at day 15.

Similar to serum concentrations, T4 and T3 levels in liver were unaffected in MAA mice compared to WT mice at both 4 and 18 weeks of age (Fig 4E and 4F). However, D1 activity was strongly decreased in 18-week old MAA mice (Fig 4G). D3 activities were hardly detectable in MAA mice and littermates (data not shown).

To investigate at which level the changes in deiodinase activities originate, we measured mRNA levels of *Dio1* and *Dio3* in the progeroid models using RT-qPCR. The results suggest that the changes in D1 and D3 activities are mainly regulated at the transcriptional level although changes in mRNA stability are not excluded (S5 Fig).

The findings in the progeroid mice prompted us to examine deiodinase expression and activity in aging WT animals. A dramatic induction (>30-fold) in *Dio3* mRNA expression was noticed in livers from 130 weeks *vs*. 13 weeks old mice (Fig 4H). D3 activity was also increased with advancing age (Fig 4I). The smaller induction of D3 activity in 104-week old mice compared to *Dio3* mRNA in 130-week old mice suggests further increased *Dio3* expression between 104 and 130 weeks of age. D1 activity showed a reciprocal decrease in aging WT mice (Fig 4J), with a significant negative correlation between the two deiodinase activities (Spearmans’s rho –0.51; *P* = 0.05).

As TH transporters and TRs are part of the TH signaling pathway, we analyzed relevant liver TH transporters and both TR isoforms in livers of all animal models (S6 Fig). No pronounced changes in transporters and receptors were observed in any of the models. Only in MAA mice, we observed a slight decrease in *Lat2* expression and a modest decrease in *Thrb* expression.

Taken together, these findings provide evidence that TH signaling in livers of rapidly and normally aging animals is reduced. As transporters and receptors were generally unaffected in contrast with the pronounced changes in D1 and D3 activity, we further focused on changes in deiodinase activities.

**Fig 5:**
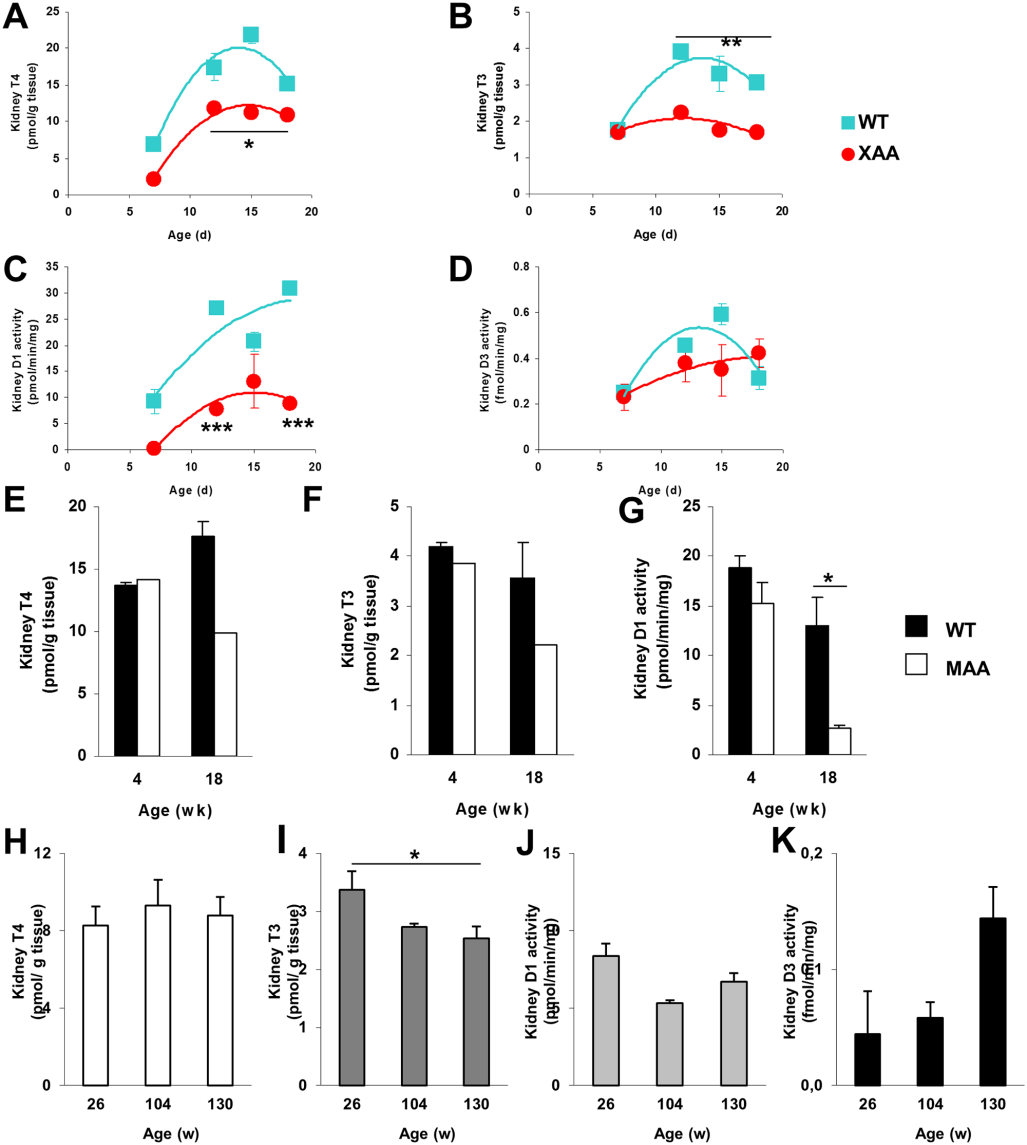
Thyroid state in kidney of progeroid and naturally aged mice. T4 (A) and T3 (B) concentrations in kidneys of 7-, 12-, 15-, and 18-day-old WT (squares) and XAA (Csbm/m/Xpa-/-) (circles) mice (n = 3/group; each time point of Csbm/m/Xpa-/- mice represents pooled tissues). Activities of D1 (C) and D3 (D) kidneys of 7-, 12-, 15-, and 18-day-old WT and XAA (Csbm/m/Xpa-/-) mice (n = 3/group). T4 (E) and T3 (F) concentrations in kidneys of 4-, and 18-week-old WT (black bars) and MAA (Ercc1-/Δ-7) (white bars) mice (n = 3/group; each time point of Ercc1-/Δ-7 mice represents pooled tissues, precluding statistical analysis). T4 (H) and T3 (I) concentrations and D1 (J) and D3 (K) activities in kidneys of 26-, 104-, and 130-week-old WT mice (n = 4-5/group). Values represent mean ± SE per group. * P < 0.05; ** P < 0.01; *** P < 0.001; # P = 0.051.

We also examined the effects of aging on TH homeostasis in the kidney. Renal T4 and T3 contents were lower in XAA mice than in age-matched WT mice, corresponding to the changes observed in serum and liver (Fig 5A and 5B). Again, substantially lower D1 expression and activity were observed in XAA vs. WT mice (Fig 5C and S7 Fig). However, in contrast to the liver, D3 did not increase with age in XAA mice, although the decline noted in WT mice at 18 days was not observed in XAA mice (Fig 5D and S7 Fig), indicating a relative increase in D3 activity in the progeroid mutant. Absolute D3 activity was ~3-fold lower in kidney than in liver, suggesting that this enzyme is less important for local TH homeostasis in kidney. These findings agree with previous observations showing that kidney TH levels mainly depend on concentrations in serum [22].

T4 and T3 levels in kidneys of MAA mice tended to be lower with advancing age, although not statistically significant (Fig 5E and 5F). The decreased D1 expression and activity over time in MAA mice is consistent with this finding (Fig 5G and S6 Fig). D3 activity was virtually undetectable in MAA and WT animals at 5 and 18 weeks of age (data not shown). Renal T3 levels decreased in old WT mice, whereas T4 levels did not change (Fig 5H and 5I). No changes in D1 activity were observed (Fig 5J). D3 activity was not significantly increased in kidneys at 130 weeks of age like in liver (Fig 5K).

### Thyroid state in heart and skeletal muscle

**Fig 6:**
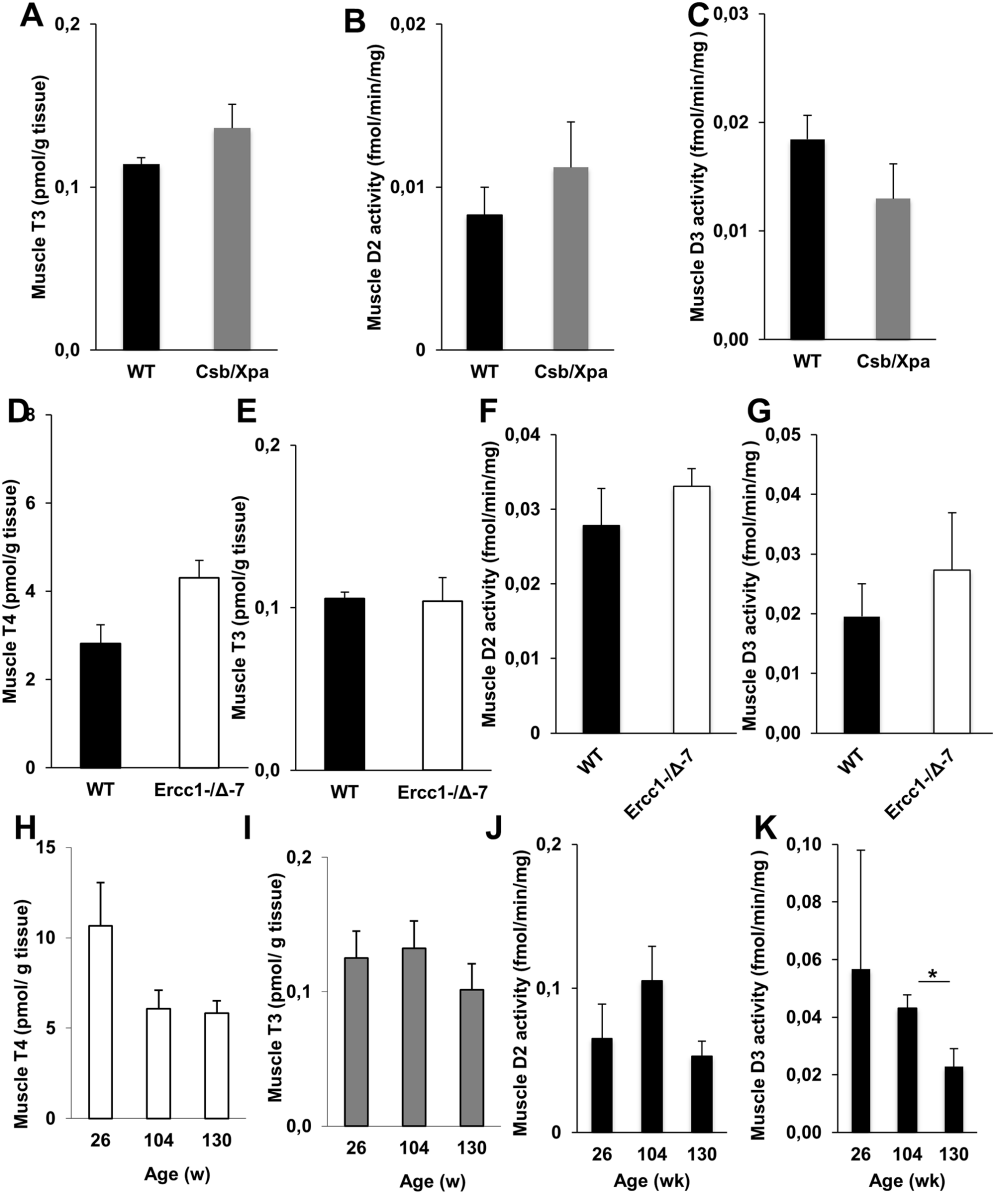
Thyroid state in skeletal muscle of progeroid and naturally aged mice. T3 concentrations (A) and activities of D2 (B) and D3 (C) in muscle of 15-day-old WT and XAA (Csbm/m/Xpa-/-) mice (n = 3/group). T4 (D) and T3 (E) concentrations and activities of D2 (F) and D3 (G) in muscle of 18-week-old WT and MAA (Ercc1-/Δ-7) mice (n = 3/group). T4 (H) and T3 (I) concentrations and activities of D2 (J) and D3 (K) in muscle of 26-, 104-, and 130-week-old WT mice (n = 3-5/group). Values represent mean ± SE per group. * P < 0.05

Given the profound changes in liver and kidney, we wondered if aging changes TH signaling in other tissues as well, especially since TH signaling can be modulated in a tissue-specific manner. TH metabolism appears remarkably preserved in muscle, since TH levels and the D2 and D3 activities expressed in muscle did not differ significantly between XAA, MAA and WT mice (Fig 6A–6G). This is in agreement with findings in normally aging mice, except for a modest decrease in D3 activity in 130-week old mice (Fig 6H–6K). Also, expression of muscle-specific T3-target genes did not change in normal or advanced aging (data not shown).

**Fig 7:**
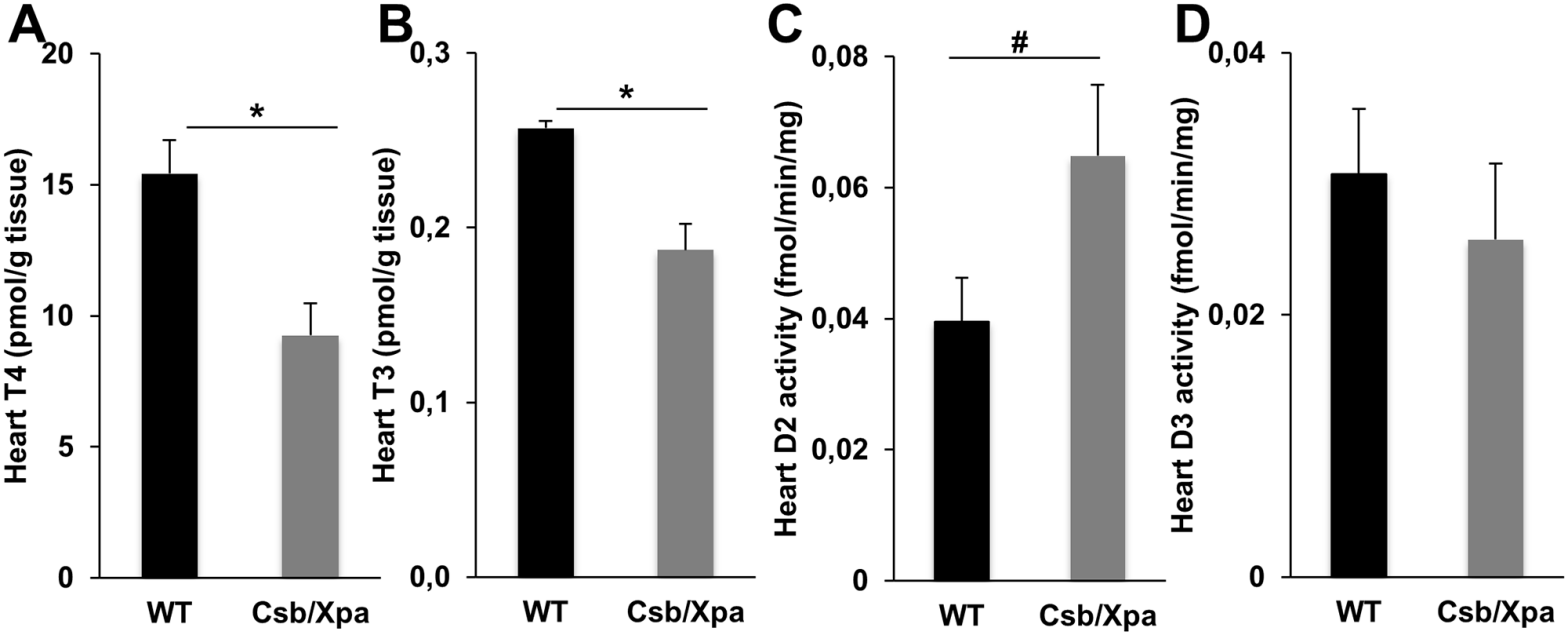
Thyroid state in heart of progeroid mice. T3 (A) and T4 (B) concentrations and activities of D2 (C) and D3 (D) in heart of 15-day-old WT and XAA (Csbm/m/Xpa-/-) mice (n = 3/group). Values represent mean ± SE. * P < 0.05; # P = 0.07

Cardiac T4 and T3 levels were decreased in XAA mice, although the T3 change was less obvious (Fig 7A and 7B). This is in line with the increase in cardiac D2 activity in these mutants, possibly in response to the lower T4 levels to ensure adequate T3 levels (Fig 7C). Cardiac D3 activity was not different between XAA and WT mice (Fig 7D). Cardiac D2 and D3 activities were barely detectable in MAA and both young and old WT mice (data not shown). Expression of heart-specific T3-target genes was not different between the different mouse models of normal and accelerated aging (data not shown).

### Thyroid state appears preserved in brains of progeroid mice

The decreased thyroid state in liver and kidney in premature and normal aging may be a beneficial response to reduce metabolism and thus to generate less reactive (damaging) metabolic by-products. On the other hand, reduced metabolism in brain might have an adverse impact given the essential role of the brain in coordinating vital functions, particularly under conditions of stress. Therefore, we sought to determine the thyroid state in brains of the different models of normal and advanced aging.

**Fig 8:**
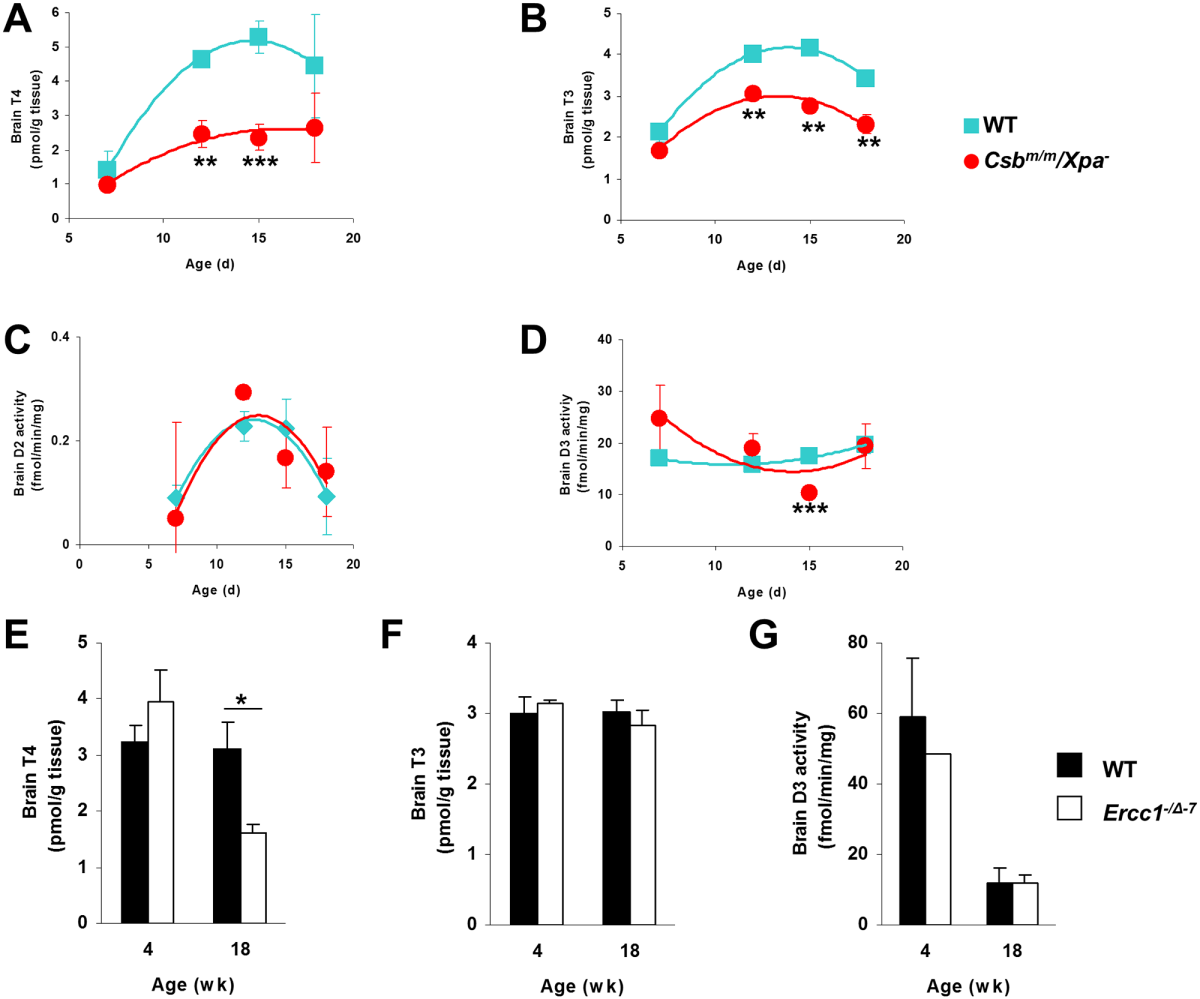
Thyroid state in brains of progeroid and naturally aged mice. Homogenates of whole brain or hemispheres were used. T4 (A) and T3 (B) concentrations in brains of 7-, 12-, 15-, and 18-day-old WT (squares) and XAA (Csbm/m/Xpa-/-) mice (n = 3/group). Activities of D2 (C) and D3 (D) brains of 7-, 12-, 15-, and 18-day-old WT and XAA (Csbm/m/Xpa-/-) mice (n = 3/group). T4 (E) and T3 (F) concentrations and D3 activity (G) in brains of 4-, and 18-week-old WT (black bars) and MAA (Ercc-/Δ-7) (white bars) mice (n = 3/group). It was not possible to measure D2 activity due to technical constraints. Values represent mean ± SE per group. * P < 0.05; ** P < 0.01; *** P < 0.001.

Both T4 and T3 content were reduced in brains of XAA mice (Fig 8A and 8B). However, in discordance with liver and kidney, the brain T3/T4 ratio tended to increase in the mutants (1.35±0.19 *vs*. 0.86±0.09; *P*<0.05). Furthermore, the D2 and D3 activities expressed in brain were similar in WT and mutant animals at most ages (Fig 8C and 8D), although D3 activity was reduced at day 15 in XAA mice (*P*<0.001) in striking contrast to the liver and the kidney.

Brain T4 levels in MAA mice decreased with age, while T3 levels were maintained (Fig 8E and 8F). In agreement with the findings in XAA mice, the brain T3/T4 ratio was increased in MAA *vs*. WT mice at 18 weeks (1.78±0.27 *vs*. 1.00±0.10 *P* = 0.05). Fig 8G shows that D3 activity did not differ between MAA and WT mice.

It is known that TH effects differ among various brain regions [32]. Because we used whole brain homogenates in the previous experiments, region-specific changes in TH signaling may have been missed. Therefore, we analyzed the expression of several well-known T3-target genes, such as *Mbp2*, *Reln*, *Bteb*, *Ptgds* and *Hr*, in the hippocampus, cerebellum and cerebral cortex of 16-week old MAA mice. Most genes were unaffected, suggesting that a euthyroid state is maintained in the MAA mouse brain (S3 Table).

XAA mice display severe growth retardation from an early postnatal stage (S8 Fig). Liver and kidney of WT animals show a continuous increase in weight during the first weeks of life (S8 Fig). In contrast, liver and kidney of XAA mice failed to show significant growth, and tissue weight was even lower at day 18 than at day 15. In contrast, absolute brain weight in XAA mice was only slightly lower, and showed a similar growth pattern as in WT mice (S8 Fig). In fact, brain weight relative to total body weight in XAA mice was even more than twice that in WT animals at day 18 (S4 Table). Thus, decreased TH metabolism in liver and kidney was associated with decreased weight of these tissues, whereas preserved TH signalling in brain was associated with relatively high brain weight.

### Interaction of GH/IGF1 signaling with D3 activity

**Fig 9:**
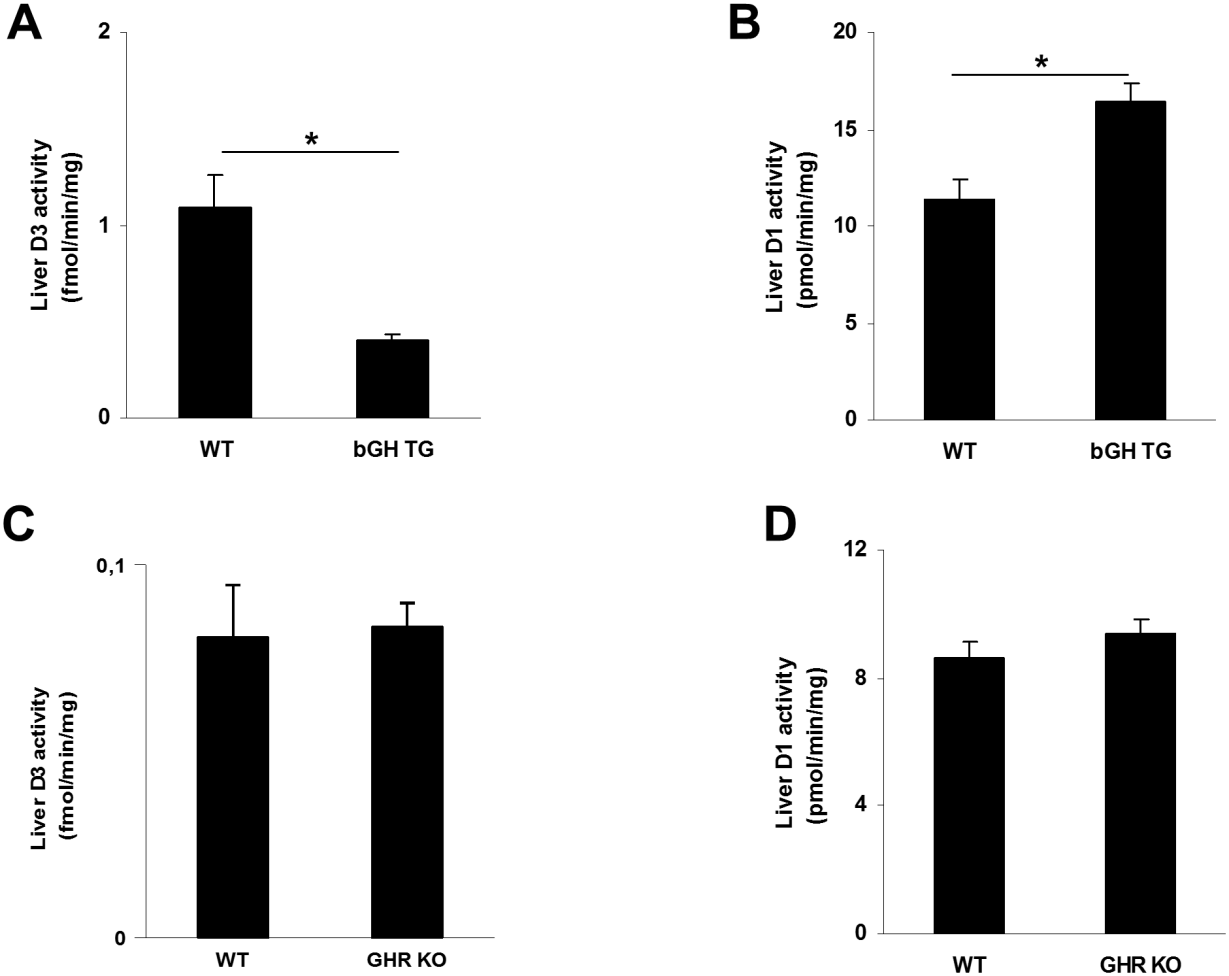
Effects of the changes in the GH/IGF1 axis on deiodinase activities. Activities of D3 (A) and D1 (B) in 2-month-old WT and bGH transgenic mice and of D3 (C) and D1 (D) in 6-month-old WT and Ghr KO mice. Values represent mean ± SE per group (n = 5–6). * P < 0.01.

In the embryonic chicken liver, GH administration induces an acute decrease in *Dio3* expression [33,34]. Since the GH/IGF1 axis is suppressed in progeroid and normally aging animals [10,12], we investigated if the aging-induced hepatic *Dio3* expression (Fig 4D and 4H) is caused by the suppressed somatotrophic axis. To test this possibility, we first assayed hepatic D3 activity in 6-month old transgenic mice over-expressing bovine GH (bGH TG mice), which exhibit shortened lifespan and several features of accelerated aging [18]. Fig 9A suggests that the GH/IGF1 pathway impinges on liver D3 activity, as this was significantly reduced in bGH TG animals compared to normal littermates. Consistent with these findings, D1 activity was slightly but significantly higher in bGH TG than in WT mice (Fig 9B). Next, we determined D1 and D3 activities in *Ghr* KO mice as a model for reduced GH/IGF1 signaling. However, overall no significant changes were detected (Fig 9C and 9D). Thus, activation of the GH/IGF1 pathway appears to suppress hepatic D3 activity, but interruption of the GH/IGF1 pathway has no effect on liver D3. Therefore, the re-activation of liver D3 in the different aging mouse models appears largely independent of the suppressed somatotrophic axis.

### WT animals treated with the pro-oxidant DEHP have lowered thyroid state

**Fig 10:**
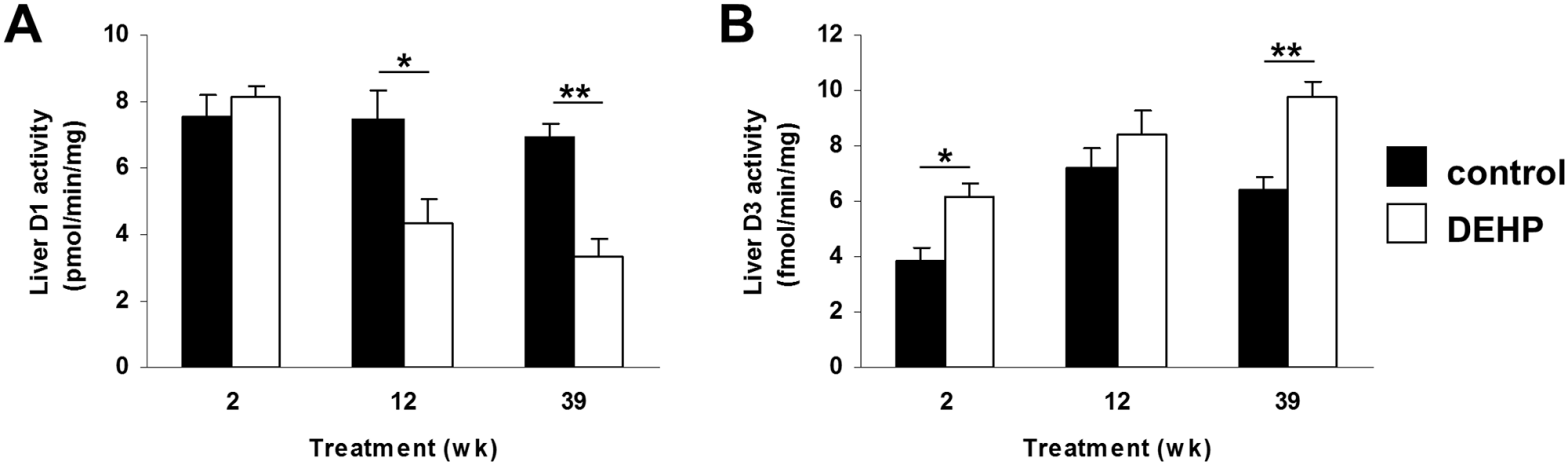
Liver D1 and D3 activity in DEHP-treated WT mice. Activities of D1 (A) and D3 (B) in 10-wk-old WT animals after exposure or not to subtoxic doses of the pro-oxidant DEHP for 2, 12 and 39 weeks. Values represent mean ± SE per group (n = 5). * P < 0.05; ** P < 0.01.

Our data from DNA repair deficient animals suggests that accumulating DNA damage somehow leads to the induction of hepatic and renal D3 activity. This hypothesis was tested using WT animals chronically exposed to oxidative stress, resulting in increased DNA damage. Therefore, WT mice were exposed through the diet for 2, 12 or 39 weeks to low levels of bis(2-ethylhexyl)phthalate (DEHP), which induces oxidative damage among others to DNA. Neither body or liver weight nor food intake of DEHP-exposed animals differed from untreated controls (data not shown), indicating that the DEHP levels used were non-toxic. As shown in Fig 10A, D1 activity was significantly and progressively decreased in DEHP-treated mice. Conversely, D3 activity was induced in mice exposed to oxidative stress (Fig 10B) in support of our hypothesis. These changes are apparently regulated at the transcriptional level since expression levels of *Dio1* were reduced and those of *Dio3* were induced (S9 Fig). Both decreased D1 activity and increased D3 activity presumably contribute to the hypothyroid state of the liver, reflected by the decreased hepatic *Dio1* and *Thsrp* expression (S9 Fig). These findings provide strong support for the hypothesis that accumulation of DNA damage underlies the suppression of TH signaling in the different models of premature and normal aging.

## Discussion

It has been well recognized that thyroid state changes during aging, but cause and consequences have remained largely elusive [35]. Since TH is one of the major stimulators of metabolism, changes in thyroid state may represent an important link between aging and key alterations in metabolism.

In the present study we used different approaches to investigate TH signaling in different models of accelerated and normal aging. First, microarray analysis of liver tissue revealed a common transcriptomic signature between hypothyroid WT mice and mice undergoing normal or accelerated aging. Similar approaches have previously demonstrated system-wide similarities between progeroid and normally aging mice [3,4]. Second, we analyzed the expression of a set of T3-responsive genes, which confirmed that livers from progeroid mutants are in a hypothyroid state. Third, we assayed hepatic D1 activity which plays a role in the peripheral conversion of T4 to bioactive T3. D1 is also positively regulated by T3, and regarded as one of the most specific markers of hepatic thyroid status [36]. In livers from XAA and MAA mice as well as normally aging mice, D1 activity was suppressed. Altogether, our findings indicate that liver TH signaling is attenuated in aging animals.

The significant decrease in expression of T3-responsive genes, including *Dio1*, in livers from MAA mice, is in contrast with the normal T3 and T4 levels in serum and liver. Several scenarios may explain this paradox. First, actions of TH are mediated by the free TH concentrations, while measured TH levels reflect total concentrations, mostly bound to carrier proteins. Therefore, the measured serum total TH concentrations may be an overestimation of the actual available TH levels. Second, TH in the liver may be sequestered to varying extents in cell compartments, limiting the availability of the hormone for the nuclear T3-receptor. Third, downregulation of Thrb in livers from MAA mice can contribute to diminished signaling at the receptor level. In contrast to liver, renal TH concentrations were decreased in MAA mice, consistent with the local modulation of effective TH concentrations (Fig 5F). Of note, compared to XAA and normally aging mice, MAA mice show dramatic features of premature aging specifically in the liver as well as in the kidney. Liver pathology includes early and massive nuclear polyploidy and invaginations, reduced proliferation, increased apoptosis, and elevated serum levels of liver-specific enzymes, pointing to severe liver pathology [3, 7, 17, 37]. Kidney aging pathology also includes karyomegaly, as well as proteinuria and progression to end stage renal failure with uremic encephalopathy [38,39]. Additionally gene-expression profiles are consistent with accelerated aging [40]. It is possible that the additional defect in interstrand cross-link repair in MAA (*Ercc1*^*-/Δ-7*^) mice results in a more pronounced hypothyroid state in the liver and to a somewhat lesser extent kidney compared to the XAA (*Csb*^*m/m*^*/Xpa*^*-/-*^) mice which lack the interstrand cross-link repair defect.

Perhaps one of the most intriguing findings in our study is the large induction of liver D3 in XAA and normally aging mice (Fig 5D, 5H and 5J). Regulation of D3 is complex and differently regulated among various tissues [13]. Normally, D3 is highly expressed in embryonic tissues and at lower levels in the adult brain, but it is hardly expressed in most other adult tissues [41]. The induction of liver D3 activity may only be partially explained by the attenuated GH/IGF1 axis in aging animals. In bGH TG animals, D3 activity was diminished, which is reminiscent of studies in which GH administration to chicken embryos acutely repressed D3 activity with a consequent increase in serum T3 levels [33,34]. Interestingly, bGH TG mice display enhanced growth but also several features of accelerated aging, including increased cancer susceptibility, and shortened lifespan [9,42]. However, only minor changes in D3 activity were observed in *Ghr* KO animals (Fig 9A and 9C), which is in line with observations of similar increases in D3 activity induced by caloric restriction in normal and *Ghr* mutant chicken [43]. Thus, it appears unlikely that the suppressed GH/IGF1 axis in progeroid mice is a major factor in the stimulation of D3 activity. Also, other factors known to interfere with TH signaling (e.g. glucocorticoid or adrenergic signaling) are unlikely to cause deiodinase changes in the progeroid and normal aging models [5,6]. Recently, several studies have unequivocally demonstrated that D3 is re-activated in certain pathological situations such as critical illness, ischemic disease, tumorigenesis, and tissue regeneration [25,44–46]. Consequently, TH inactivation is increased in these conditions, resulting in lower TH levels. Interestingly, induction of D3 can affect TH concentrations in a tissue-specific way without impeding thyroid state systemically [13]. Our results clearly demonstrate that aging is also associated with induction of D3 expression in particular in liver. It is important to note that this occurs in the absence of obvious cachexia or illness. Hepatic D3 activity was induced not only in normally and rapidly aging mice but also in WT mice after chronic exposure to sub-toxic concentrations of the pro-oxidant DEHP, all animal models associated with increased DNA damage. In addition, all models displayed reduced liver D1 activity. Although submicroscopic abnormalities cannot be excluded, histological examination of the progeroid thyroid glands was unremarkable. Also, the pituitary appears unaffected, which is in line with previous reports [3,4]. Thus, changes in peripheral deiodination, including the increased D3-mediated T3 and T4 inactivation and decreased D1-mediated T3 production, may contribute to the diminished TH signaling during aging.

As noted above in the context of Ercc1 mutant mice, also the kidneys appeared in a hypothyroid state in the different models of normal and accelerated aging, although less pronounced than in the liver. Except for normally aging mice, no induction of D3 was observed. Given the 3-fold lower activity in kidney *versus* liver, D3 may be less important for local TH signaling in the kidney.

In contrast to liver and kidney, TH signaling appears not to be changed in heart and skeletal muscle during aging. If anything, the up-regulation of cardiac D2 in XAA mice, and the down-regulation of muscle D3 in normally aging mice may even suggest preserved TH signaling in these tissues. Indeed, muscle and liver show a divergent response in TH signaling during illness [47]. In both mutant models, no change in TSH was observed, which might reflect a relative insensitivity of the pituitary gland towards TH. However, in WT aging animals we noticed a small but significant increase in TSH levels in the pituitary at 130 weeks of age. This supports the idea that aging brings about a lowering of thyroid state.

Also in brain TH signaling appears to be preserved. Although TH levels and deiodinase activities were measured in whole brain, while TH homeostasis may be controlled in a region-specific manner, our findings do not support the idea of a global decrease in TH signaling in the aging brain. In fact, the observation that the T3/T4 ratio tended to increase in the brain, rather than to decrease as in the liver, is compatible with the maintenance of vital brain functions during aging. This is also suggested by the observation that brain weight is clearly less decreased than total body, liver and kidney weight in XAA mice. Interestingly, in various species caloric restriction results in larger reductions in liver weight than in brain weight [48,49]. Thus, progeroid mutants appear to reallocate resources and metabolism to spare the most vital organs, suggesting that preserved functional brain capacity is beneficial for survival.

All aspects of TH signaling showed similar trends in normally vs. rapidly aging mice. The most prominent changes were noted in XAA mice the most severe progeroid model in our studies. This is conceivable as lifespan is dramatically reduced in these animals (3–5 weeks) compared to WT mice and even MAA mice (up to 6 months). Therefore, the severity of the progeroid phenotype may correlate with the degree of the TH response to aging [2,3,5]. Although changes were less pronounced in normally aging animals, the strong similarities with changes observed in the progeroid mice indicate that the latter provide good models for normal aging. DNA damage may induce different responses in different tissues, because of varying efficacies of mechanisms to induce, prevent or repair such damage, which may explain differences between the different models of normal and advanced aging examined. Nevertheless, our findings suggest that a common mechanism affects the cellular deiodinase activities in normal and premature aging. Future studies in mouse models with other DNA repair defects will establish if the present findings are universal.

**Fig 11:**
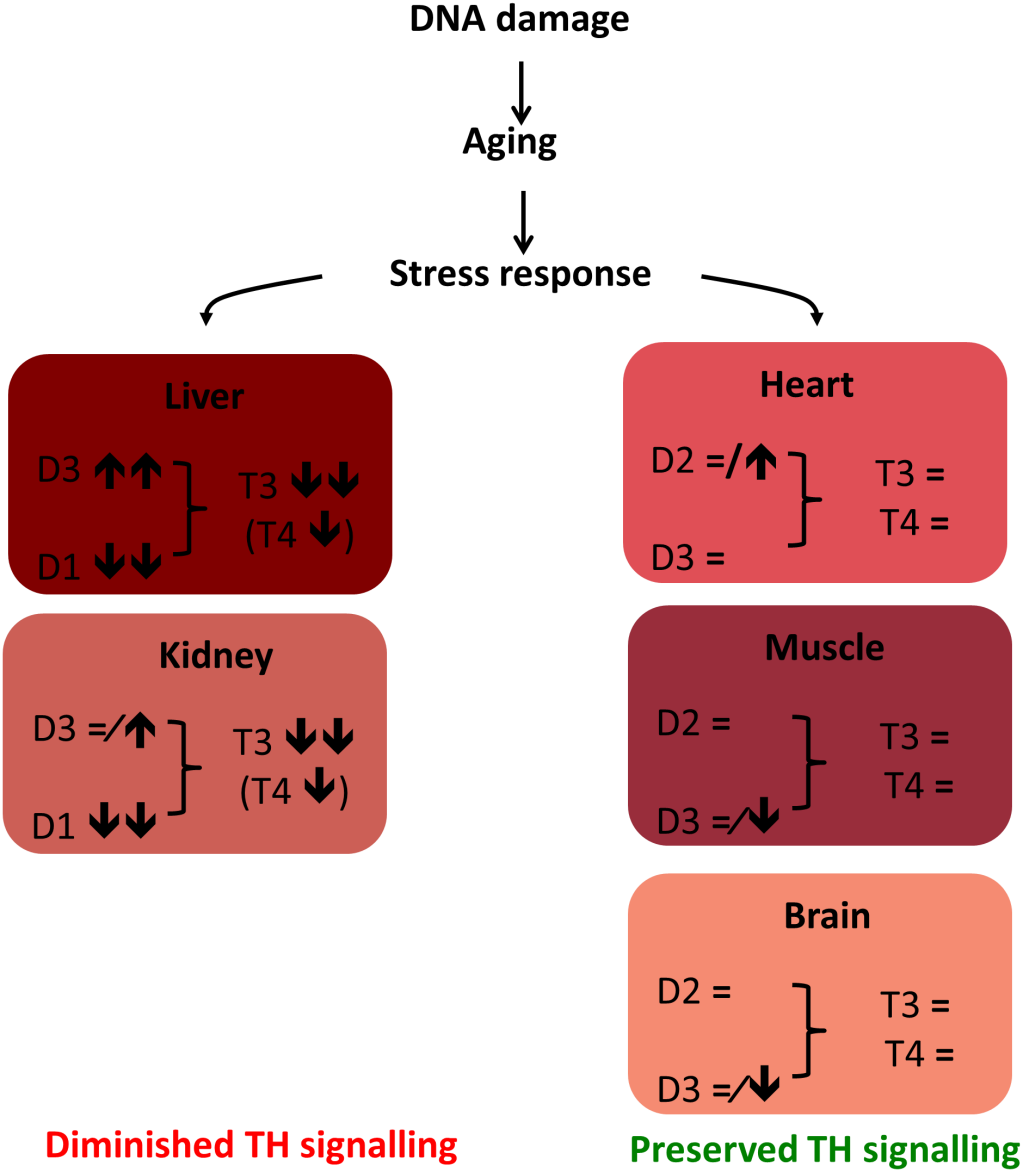
Schematic representation of the survival response. Several types of stress (e.g. DNA damage and aging) can trigger a differential response in various tissues. This response ensures decreased TH signalling in liver and kidney, while it preserves TH signalling in brain, muscle and heart.

What is the relevance of attenuated TH signaling during aging? Previous investigations have reported reduced GH/IGF1 signaling during aging [2–4]. Because suppression of the somatotrophic axis is also observed in stress situations such as calorie restriction or disease, it is argued that the consequent reduction in growth and metabolism (and up-regulation of defense mechanisms including the anti-oxidant system) reflects an attempt to survive stressful conditions [5]. This suppression of the GH/IGF1 axis may be regarded as an adaptive ‘survival’ response which is triggered also during DNA damage accumulation and the aging process [5]. At present, the exact underlying molecular mechanisms which initiate this trigger are unknown. Our observations are reminiscent of changes in thyroid state during periods of stress [50]. Dwarf mice with pituitary malfunction resulting in lower GH, IGF1 and TH concentrations also have a prolonged lifespan, whereas life-long T4 substitution of these animals markedly shortens their lifespan [14–16]. Thus, moderate hypothyroidism may prolong lifespan in mice [51]. These observations suggest that a global reduction in TH-driven metabolism functions as a protective mechanism to survive a stressful period (Fig 11). Apparently, less vital organs (liver, kidney) benefit from decreased TH signaling, while in other organs (brain, muscle, heart) it is essential to maintain normal TH metabolism. Thus, attenuated TH signaling in liver and kidney likely represents part of a protective survival response elicited in normal and in premature aging due to defective DNA repair, which is highly tissue-specific.

What is the relevance of our findings for humans? Several reports suggest that subclinical hypothyroidism in elderly people is associated with increased lifespan [52,53]. Furthermore, genetic predisposition for a subtle decrease in thyroid function is associated with increased longevity in centenarians and their offspring [54,55]. In addition, several studies suggest that subclinical hypothyroidism provides some functional advantage in daily living tasks [53,56]. This may have important clinical implications for the decision whether or not to treat elderly patients with mild hypothyroidism. Our results suggest that the decreased thyroid state during aging is an adaptive response, providing a mechanistic basis for the epidemiological observations in humans. Future studies should explore further underlying mechanisms as well as gender differences (which was not assessed in the present study) in the response to DNA damage.

Until now, the molecular relationship between DNA damage, aging and changes in thyroid state were elusive. The present study for the first time provides evidence that DNA damage may contribute to the attenuated TH signaling during aging. In addition, we discovered a novel role of D3 during the aging process. We propose that the tissue-specific regulation of deiodinase activities resulting in decreased TH signaling in particular tissues importantly contributes to the adaptive survival response during aging both at the systemic and organ-specific level. This response likely aims to minimize further damage by an evolutionary strongly selected reduction and redesign of metabolism and shift from growth and proliferation to a preservation mode.

## Supporting Information

S1 Fig Differentially expressed genes of all models. (PPT)

S2 Fig Pathways of differentially expressed genes. (PPT)

S3 Fig Microarray expression of selected T3-responseive gens. (PPT)

S4 Fig TSHb expression levels in pituitary. (PPT)

S5 Fig Dio1 and Dio3 expression levels in livers of Csb/Xpa DKO mice. (PPT)

S6 Fig Expression levels of TH transporters and TRs in Csb/Xpa DKO mice, Ercc1-/Δ-7 mice and naturally aging mice. (PPT)

S7 Fig Dio1 and Dio3 expression levels in kidneys of Csb/Xpa DKO mice. (PPT)

S8 Fig Body and tissue weights of Csb/Xpa DKO mice. (PPT)

S9 Fig Deiodinase activities in bGH transgenic and Ghr KO mice. (PPT)

S1 Table Primer sequences for qPCR. (XLS)

S2 Table Differentially expressed genes of all models. (XLS)

S3 Table T3-responsive genes in brain of Ercc1-/Δ-7 mice. (XLS)

S4 Table Body weights of Csb/Xpa DKO mice. (XLS)
