# Supplementary material for: Tissue-Specific Suppression of Thyroid Hormone Signaling in Various Mouse Models of Aging
Source: PLoS One. 2016 Mar 8;11(3):e0149941. doi: 10.1371/journal.pone.0149941 (PMC4783069; doi:10.1371/journal.pone.0149941)
Supplement: S1 Fig — (PPT) [file pone.0149941.s001.ppt]

## Slide 1
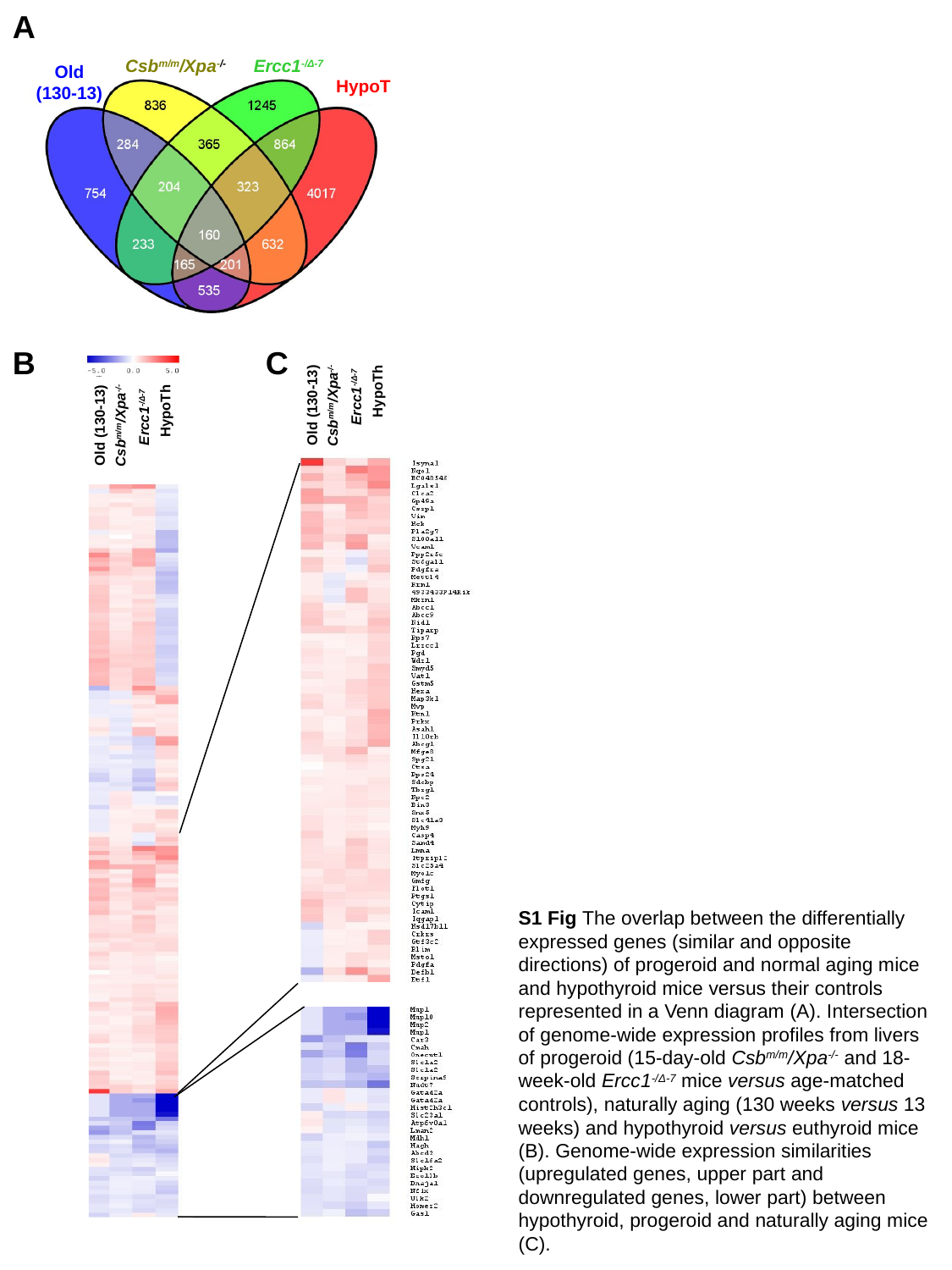

A
Csbm/m/Xpa-/-
Ercc1-/Δ-7
Old (130-13)
HypoT
B
C
HypoTh
Old (130-13)
HypoTh
Csbm/m/Xpa-/-
Ercc1-/Δ-7
Old (130-13)
Csbm/m/Xpa-/-
Ercc1-/Δ-7
S1 Fig The overlap between the differentially expressed genes (similar and opposite directions) of progeroid and normal aging mice and hypothyroid mice versus their controls represented in a Venn diagram (A). Intersection of genome-wide expression profiles from livers of progeroid (15-day-old Csbm/m/Xpa-/- and 18-week-old Ercc1-/Δ-7 mice versus age-matched controls), naturally aging (130 weeks versus 13 weeks) and hypothyroid versus euthyroid mice (B). Genome-wide expression similarities (upregulated genes, upper part and downregulated genes, lower part) between hypothyroid, progeroid and naturally aging mice (C).
