# Supplementary material for: Tissue-Specific Suppression of Thyroid Hormone Signaling in Various Mouse Models of Aging
Source: PLoS One. 2016 Mar 8;11(3):e0149941. doi: 10.1371/journal.pone.0149941 (PMC4783069; doi:10.1371/journal.pone.0149941)
Supplement: S2 Fig — (PPT) [file pone.0149941.s002.ppt]

## Slide 1
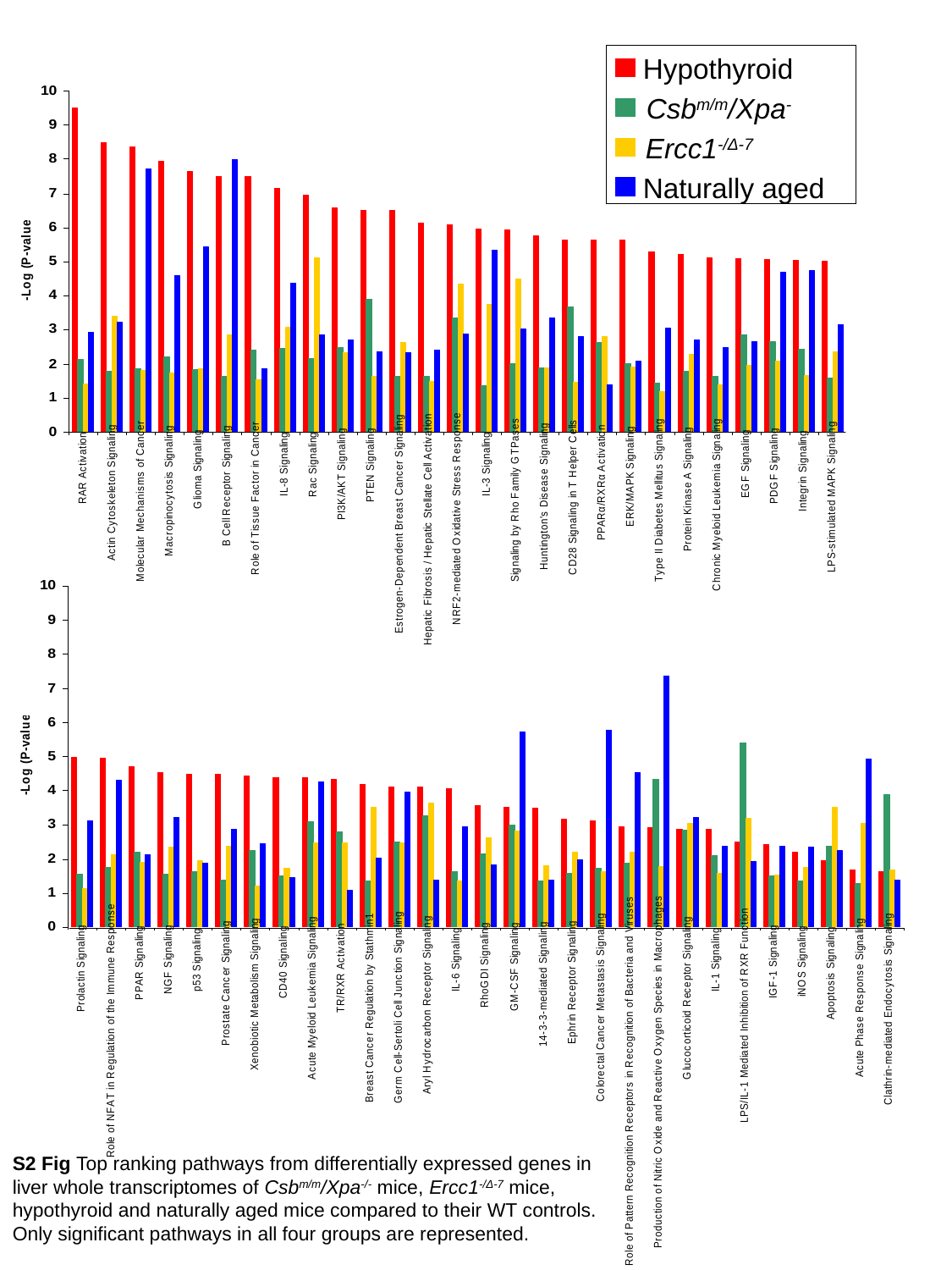

Hypothyroid
Csbm/m/Xpa-
Ercc1-/Δ-7
Naturally aged
S2 Fig Top ranking pathways from differentially expressed genes in liver whole transcriptomes of Csbm/m/Xpa-/- mice, Ercc1-/Δ-7 mice, hypothyroid and naturally aged mice compared to their WT controls. Only significant pathways in all four groups are represented.
