# Supplementary material for: Tissue-Specific Suppression of Thyroid Hormone Signaling in Various Mouse Models of Aging
Source: PLoS One. 2016 Mar 8;11(3):e0149941. doi: 10.1371/journal.pone.0149941 (PMC4783069; doi:10.1371/journal.pone.0149941)
Supplement: S3 Fig — (PPT) [file pone.0149941.s003.ppt]

## Slide 1
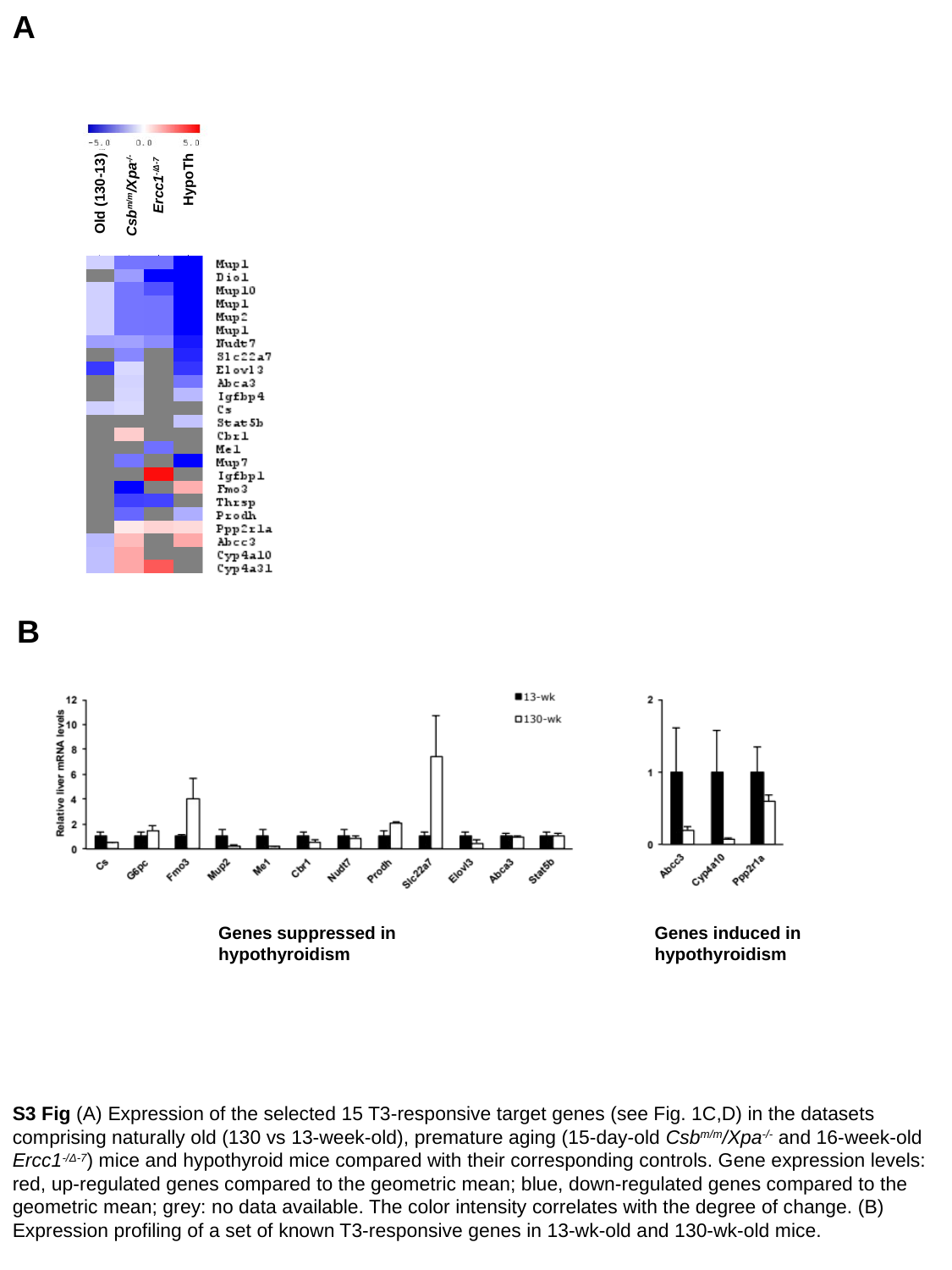

A
HypoTh
Old (130-13)
Ercc1-/Δ-7
Csbm/m/Xpa-/-
B
Genes suppressed in hypothyroidism
Genes induced in hypothyroidism
S3 Fig (A) Expression of the selected 15 T3-responsive target genes (see Fig. 1C,D) in the datasets comprising naturally old (130 vs 13-week-old), premature aging (15-day-old Csbm/m/Xpa-/- and 16-week-old Ercc1-/Δ-7) mice and hypothyroid mice compared with their corresponding controls. Gene expression levels: red, up-regulated genes compared to the geometric mean; blue, down-regulated genes compared to the geometric mean; grey: no data available. The color intensity correlates with the degree of change. (B) Expression profiling of a set of known T3-responsive genes in 13-wk-old and 130-wk-old mice.
