# Supplementary material for: Tissue-Specific Suppression of Thyroid Hormone Signaling in Various Mouse Models of Aging
Source: PLoS One. 2016 Mar 8;11(3):e0149941. doi: 10.1371/journal.pone.0149941 (PMC4783069; doi:10.1371/journal.pone.0149941)
Supplement: S4 Fig — (PPT) [file pone.0149941.s004.ppt]

## Slide 1
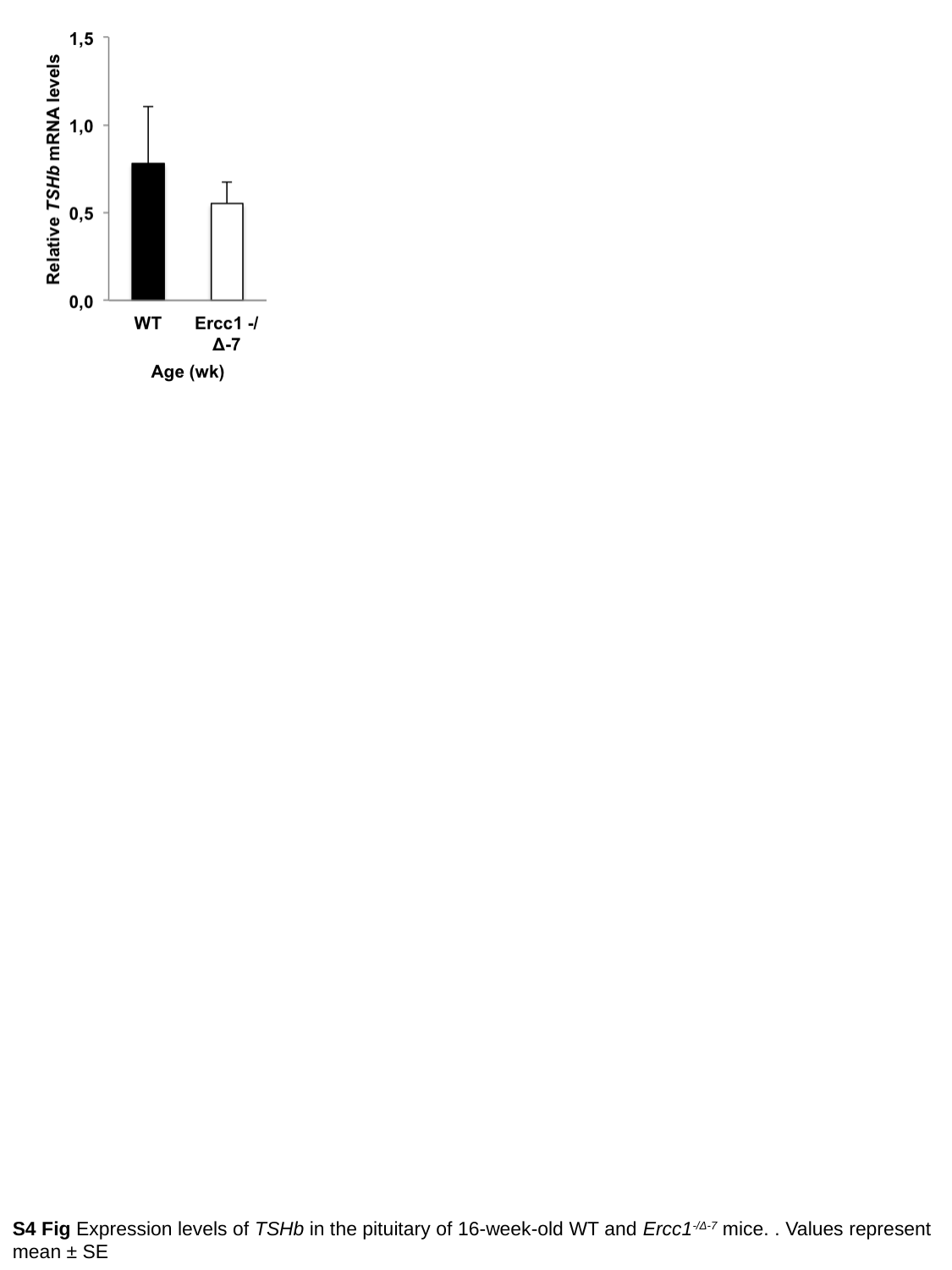

S4 Fig Expression levels of TSHb in the pituitary of 16-week-old WT and Ercc1-/Δ-7 mice. . Values represent mean ± SE
