# Supplementary material for: Tissue-Specific Suppression of Thyroid Hormone Signaling in Various Mouse Models of Aging
Source: PLoS One. 2016 Mar 8;11(3):e0149941. doi: 10.1371/journal.pone.0149941 (PMC4783069; doi:10.1371/journal.pone.0149941)
Supplement: S5 Fig — (PPT) [file pone.0149941.s005.ppt]

## Slide 1
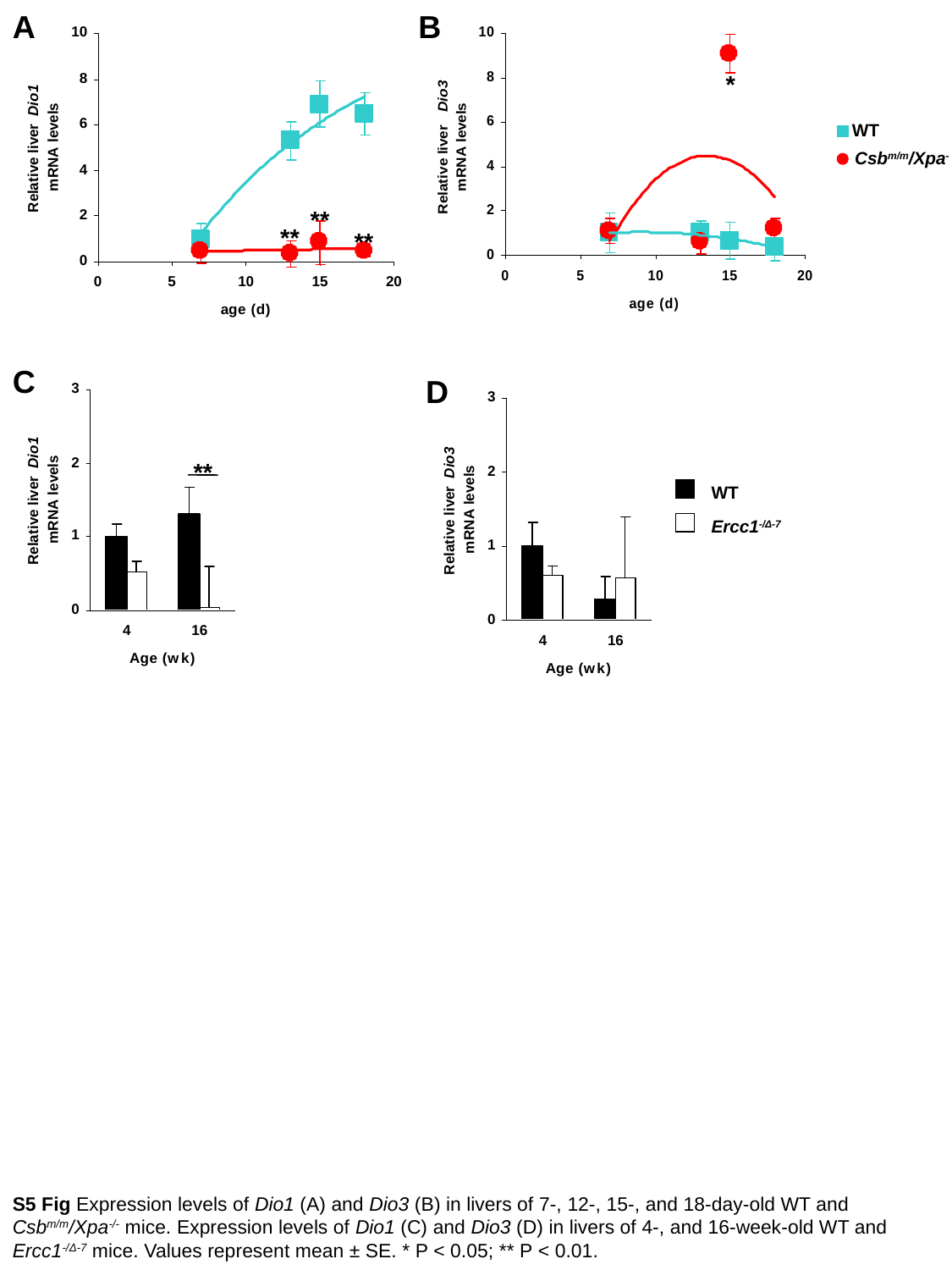

A
B
*
WT
Csbm/m/Xpa-
**
**
**
C
D
**
WT
Ercc1-/Δ-7
S5 Fig Expression levels of Dio1 (A) and Dio3 (B) in livers of 7-, 12-, 15-, and 18-day-old WT and Csbm/m/Xpa-/- mice. Expression levels of Dio1 (C) and Dio3 (D) in livers of 4-, and 16-week-old WT and Ercc1-/Δ-7 mice. Values represent mean ± SE. * P < 0.05; ** P < 0.01.
