# Supplementary material for: Tissue-Specific Suppression of Thyroid Hormone Signaling in Various Mouse Models of Aging
Source: PLoS One. 2016 Mar 8;11(3):e0149941. doi: 10.1371/journal.pone.0149941 (PMC4783069; doi:10.1371/journal.pone.0149941)
Supplement: S6 Fig — (PPT) [file pone.0149941.s006.ppt]

## Slide 1
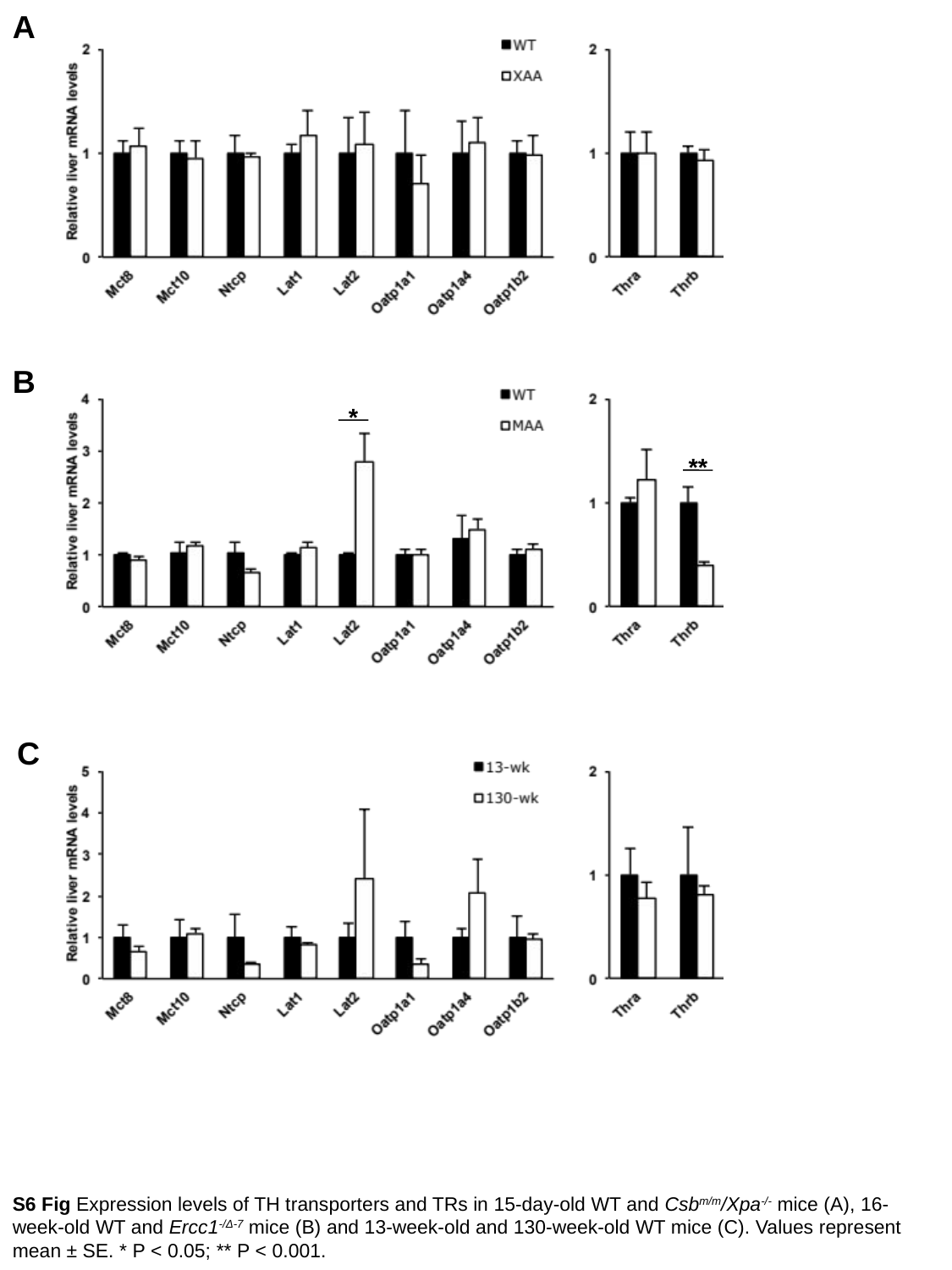

A
B
*
**
C
S6 Fig Expression levels of TH transporters and TRs in 15-day-old WT and Csbm/m/Xpa-/- mice (A), 16-week-old WT and Ercc1-/Δ-7 mice (B) and 13-week-old and 130-week-old WT mice (C). Values represent mean ± SE. * P < 0.05; ** P < 0.001.
