# Supplementary material for: Tissue-Specific Suppression of Thyroid Hormone Signaling in Various Mouse Models of Aging
Source: PLoS One. 2016 Mar 8;11(3):e0149941. doi: 10.1371/journal.pone.0149941 (PMC4783069; doi:10.1371/journal.pone.0149941)
Supplement: S8 Fig — (PPT) [file pone.0149941.s008.ppt]

## Slide 1
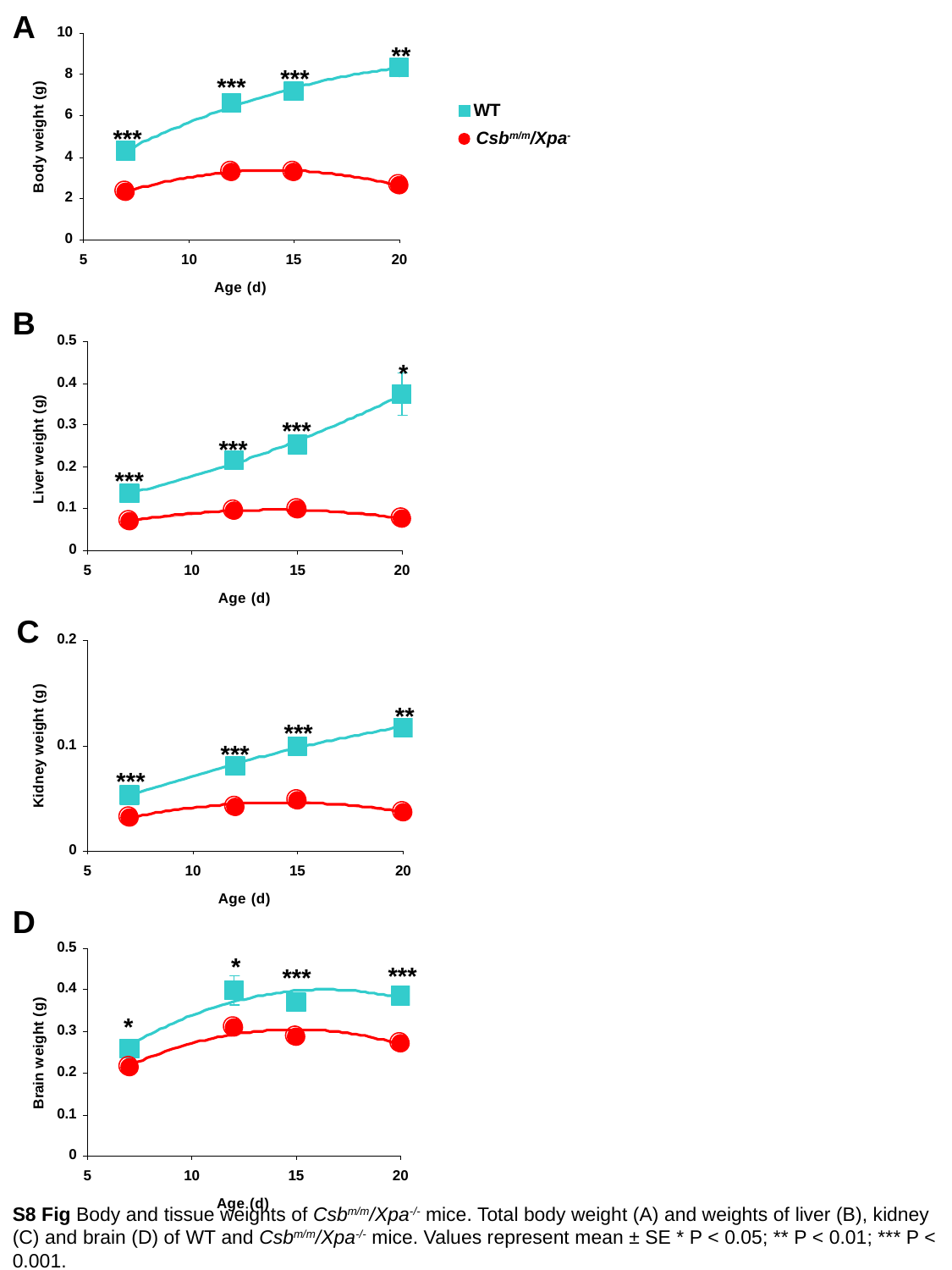

A
**
***
***
WT
Csbm/m/Xpa-
***
B
*
***
***
***
C
**
***
***
***
D
*
***
***
*
S8 Fig Body and tissue weights of Csbm/m/Xpa-/- mice. Total body weight (A) and weights of liver (B), kidney (C) and brain (D) of WT and Csbm/m/Xpa-/- mice. Values represent mean ± SE * P < 0.05; ** P < 0.01; *** P < 0.001.
