# Supplementary material for: Tissue-Specific Suppression of Thyroid Hormone Signaling in Various Mouse Models of Aging
Source: PLoS One. 2016 Mar 8;11(3):e0149941. doi: 10.1371/journal.pone.0149941 (PMC4783069; doi:10.1371/journal.pone.0149941)
Supplement: S9 Fig — (PPT) [file pone.0149941.s009.ppt]

## Slide 1
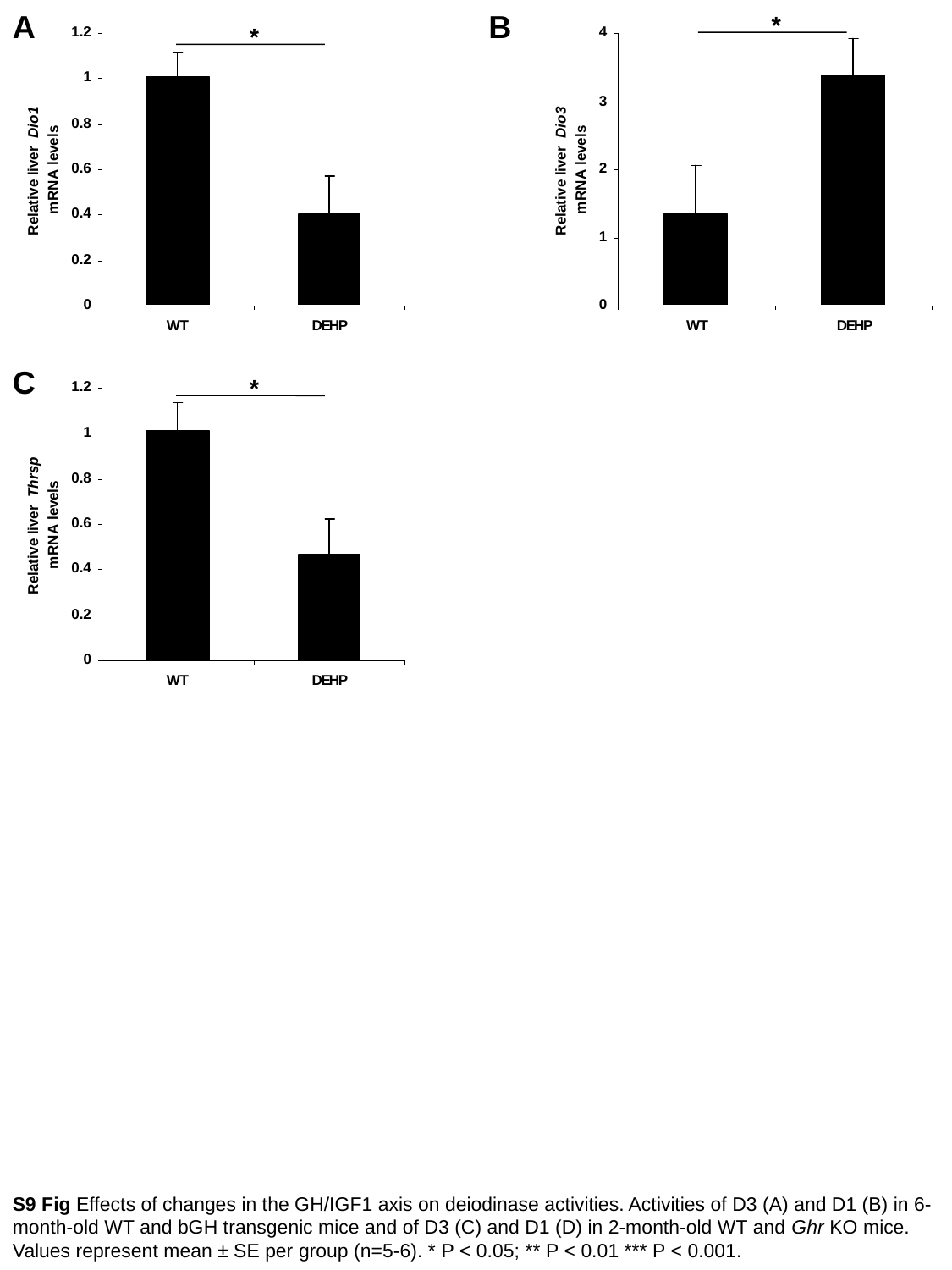

A
B
*
*
C
*
S9 Fig Effects of changes in the GH/IGF1 axis on deiodinase activities. Activities of D3 (A) and D1 (B) in 6-month-old WT and bGH transgenic mice and of D3 (C) and D1 (D) in 2-month-old WT and Ghr KO mice. Values represent mean ± SE per group (n=5-6). * P < 0.05; ** P < 0.01 *** P < 0.001.
